# Stable Tensor Principal Component Pursuit: Error Bounds and Efficient Algorithms

**DOI:** 10.3390/s19235335

**Published:** 2019-12-03

**Authors:** Wei Fang, Dongxu Wei, Ran Zhang

**Affiliations:** 1 Department of Computer Science and Technology, Huaibei Vocational and Technical College, Huaibei 235000, China; 2 School of Physics and Electronic Electrical Engineering, Huaiyin Normal University, Huaian 223300, China; weidx@hytc.edu.cn; 3 Mathematics Teaching and Research Group, Nanjing No.9 High School, Nanjing 210018, China; exjzhang@163.com

**Keywords:** tensor principal component pursuit, stable recovery, tensor SVD, ADMM

## Abstract

The rapid development of sensor technology gives rise to the emergence of huge amounts of tensor (i.e., multi-dimensional array) data. For various reasons such as sensor failures and communication loss, the tensor data may be corrupted by not only small noises but also gross corruptions. This paper studies the Stable Tensor Principal Component Pursuit (STPCP) which aims to recover a tensor from its corrupted observations. Specifically, we propose a STPCP model based on the recently proposed tubal nuclear norm (TNN) which has shown superior performance in comparison with other tensor nuclear norms. Theoretically, we rigorously prove that under tensor incoherence conditions, the underlying tensor and the sparse corruption tensor can be stably recovered. Algorithmically, we first develop an ADMM algorithm and then accelerate it by designing a new algorithm based on orthogonal tensor factorization. The superiority and efficiency of the proposed algorithms is demonstrated through experiments on both synthetic and real data sets.

## 1. Introduction

In recent years, different types of tensor data have emerged with the significant progress of modern sensor technology, such as color images [1], videos [2], functional MRI data [3], hyper-spectral images [4], point could data [5], traffic stream data [6], etc. Thanks to its multi-way nature, tensor-based methods have natural superiority over vector and matrix-based methods in analyzing and processing ubiquitous modern multi-way data, and have found extensive applications in computer vision [1,7], data mining [5], machine learning [2], signal processing [8], to name a few. In real applications, the acquired tensor data may often suffer from noises and gross corruptions owing to many different reasons such as sensor failure, lens pollution, communication interference, occlusion in videos, or abnormalities in a sensor network [9], etc. At the same time, many real-world tensor data, such as face images or videos, have been shown to have some low-dimensional structure and can be well approximated by a smaller number of “principal components” [8]. Then, a question naturally arises: how to pursue the principal components of an observed tensor data in the presence of both noises and gross corruptions? We will answer this question in this paper and refer to the proposed methodology as Stable Tensor Principal Component Pursuit (STPCP).

The tensor low-rankness is an ideal model of the property that a tensor data can be well approximated by a small number of principal components [8]. In the last decade, low-rank tensor models have attracted much attention in many fields [10]. There are multiple low-rank tensor models since there exist different definitions of tensor rank. Among these models, the low CP rank model [11] and the low Tucker rank model [1] should be the most famous two. The low CP rank model approximates the underlying tensor by the sum of a small number of rank-1 tensors, whereas the low Tucker rank model assumes the unfolding matrix along each mode are low rank. To estimate an unknown low-rank tensor from corrupted observations, it is a natural option to consider the rank minimization problem which chooses the tensor of lowest rank as the solution from a certain feasible set. However, tensor rank minimization, even in its 2-way (matrix) case, is generally NP-hard [12] and even harder in higher-way cases [13]. For tractable solutions, researchers turn to a variety of convex surrogates for tensor rank  [1,14,15,16,17,18] to replace the tensor rank in rank minimization problem. Methods based on surrogates for the tensor CP rank and Tucker rank have been extensively explored in both the theoretical side and the application side [14,17,19,20,21,22,23,24].

Recently, the low-tubal-rank model [16,25] has shown better performance than traditional tensor low-rank models in many tensor recover tasks such as image/video inpainting/denoising/ sensing [2,25,26], moving object detection [27], multi-view learning [28], seismic data completion [29], WiFi fingerprint [30], MRI imaging [16], point cloud data inpainting [31], and so on. The tubal rank is a new complexity measure of tensor defined through the framework of tensor singular value decomposition (t-SVD) [32,33]. At the core of existing low-tubal-rank models is the tubal nuclear norm (TNN) which is a convex surrogate for the tubal rank. In contrast to CP-based tensor nuclear norms or Tucker-based tensor nuclear norms which models low-rankness in the original domain, TNN models low-rankness in the Fourier domain. It is pointed out in [25,34,35] that TNN has superiority over traditional tensor nuclear norms in exploiting the ubiquitous “spatial-shifting” property in real-world tensor data.

Inspired by the superior performance of TNN, this paper adopts TNN as a low-rank regularizer in the proposed STPCP model. Specifically, the proposed STPCP aims to estimate the underlying tensor data ${\underline{L}}_{0}\in R^{n_{1}\times n_{2}\times n_{3}}$ from an observation tensor $\underline{M}$ polluted by both small dense noises and sparse gross corruptions as follows:

(1) $$\begin{array}{c} \underline{M}={\underline{L}}_{0}+{\underline{S}}_{0}+{\underline{E}}_{0}, \end{array}$$

where ${\underline{S}}_{0}$ is a tensor denoting the sparse corruptions and ${\underline{E}}_{0}$ is a tensor representing small dense noises. Model (1) is also known as robust tensor decomposition in [36,37].

Our STPCP model is first formulated as a TNN-based convex problem. Then, our theoretical analysis gives upper bound on the estimation error of ${\underline{L}}_{0}$ and ${\underline{S}}_{0}$. In contrast to the analysis in [37], the proposed STPCP can exactly recovery the underlying tensor ${\underline{L}}_{0}$ and the sparse corruption tensor ${\underline{S}}_{0}$ when the noise term ${\underline{E}}_{0}$ vanishes. For efficient solution of the proposed STPCP model, we develop two algorithms with extensions to a more challenging scenario where missing observations are also considered. The first algorithm is an ADMM algorithm and the second algorithm accelerates it using tensor factorization. Experiments show the effectiveness and the efficiency of the designed algorithms.

We organize the rest of this paper as follows. In Section 2, we briefly introduce basic preliminaries for t-SVD and some related works. The proposed STPCP model is formulated and analyzed theoretically in Section 3. We design two algorithms in Section 4 and report experimental results in Section 5. This work is concluded in Section 6. The proofs of theorems, propositions, and lemmas are given in the appendix.

## 2. Preliminaries and Related Works

In this section, some preliminaries of t-SVD are first introduced. Then, the related works are presented.

**Notations.** We denote vectors by bold lower-case letters, e.g., $a\in R^{n}$, matrices by bold upper-case letters, e.g., $A\in R^{n_{1}\times n_{2}}$, and tensors by underlined upper-case letters, e.g., $\underline{A}\in R^{n_{1}\times n_{2}\times n_{3}}$. For a given 3-way tensor, we define its fiber as a vector given through fixing all indices but one, and its *slice* as a matrix obtained by fixing all indices but two. For a given 3-way tensor $\underline{A}$, we use ${\underline{A}}_{ijk}$ to denote its (i,j,k)-th element; $A^{\left(k\right)}:=\underline{A}\left(:,:,k\right)$ is used to denote its *k*-th frontal slice. $\tilde{\underline{A}}$ is used to denote the tensor after performing 1D Discrete Fourier Transformation (DFT) on all tube fibers $\underline{A}\left(i,j,:\right)$ of $\underline{T}$, $\forall i=1,2,\cdots ,n_{1},\;j=1,2,\cdots ,n_{2}$, which can be efficiently computed by the Matlab command $\tilde{\underline{A}}=\mathrm{fft}\left(\underline{A},\left[\right],3\right)$. We use dft3(·) and idft3(·) to represent the 1D DFT and inverse DFT along the tube fibers of 3-way tensors, i.e.,  $\mathrm{dft}3\left(\underline{A}\right):=\mathrm{fft}\left(\underline{A},\left[\right],3\right),\;\mathrm{idft}3\left(\underline{A}\right):=\mathrm{ifft}\left(\underline{A},\left[\right],3\right).$

For a given matrix $M\in R^{n_{1}\times n_{2}}$, define the nuclear norm and spectral norm of M respectively as:

$$\begin{array}{c} {∥M∥}_{*}:=\sum_{i=1}^{p}\sigma_{i}\left(M\right),\;\mathrm{and}\;{∥M∥}_{\mathrm{sp}}:=\max\left\{\sigma_{i}\left(M\right)\right\}, \end{array}$$

where $p=\min\{n_{1},n_{2}\}$, and $\sigma_{1}\left(M\right)\geq \cdots \geq \sigma_{p}\left(M\right)$ are the singular values of M in a non-ascending order. The $l_{0}$-norm, $l_{1}$-norm, Frobenius norm, $l_{\infty }$-norm of a tensor $\underline{A}\in R^{n_{1}\times n_{2}\times n_{3}}$ is defined as:

$$\begin{array}{c} ∥\underline{A}{∥}_{0}:=\sum_{ijk}1\left({\underline{A}}_{ijk}\neq 0\right),\;∥\underline{A}{∥}_{1}:=\sum_{ijk}|{\underline{A}}_{ijk}|,\;∥\underline{A}{∥}_{F}:=\sqrt{\sum_{ijk}{\underline{A}}_{ijk}^{2}},\;∥\underline{A}{∥}_{\infty }:=\max_{ijk}\left|{\underline{A}}_{ijk}\right|, \end{array}$$

where 1(C) is an indicator function whose value is 1 if the condition *C* is true, and 0 otherwise.

Given two matrices $A=\left(a_{ij}\right)\in C^{n_{1}\times n_{2}},B=\left(b_{ij}\right)\in C^{n_{1}\times n_{2}}$, we define their inner product as follows:

$$\begin{array}{c} \langle A,B\rangle =\mathrm{tr}\left(A^{H}B\right)=\sum_{ij}{\bar{a}}_{ij}b_{ij}, \end{array}$$

where $A^{H}$ denotes conjugate transpose of matrix A and ${\bar{a}}_{ij}$ denotes the conjugation of complex number $a_{ij}$. Given two 3-way tensors $\underline{A},\underline{B}\in R^{n_{1}\times n_{2}\times n_{3}}$, we define their inner product as follows:

$$\begin{array}{c} \langle \underline{A},\underline{B}\rangle :=\sum_{ijk}{\underline{A}}_{ijk}{\underline{B}}_{ijk}. \end{array}$$

### 2.1. Tensor Singular Value Decomposition

We first define 3 operators based on block matrices which are introduced in [33]. For a given 3-way tensor $\underline{A}\in R^{n_{1}\times n_{2}\times n_{3}}$, we define its block vectorization bvec(·) and the inverse operation bvfold(·) in the following equation:

$$\begin{array}{c} \mathrm{bvec}\left(\underline{A}\right):=\left[\begin{array}{c} A^{\left(1\right)}\\ A^{\left(2\right)}\\ \vdots \\ A^{\left(n_{3}\right)} \end{array}\right]\;\in R^{n_{1}n_{3}\times n_{2}},\;\mathrm{bvfold}\left(\mathrm{bvec}\left(\underline{A}\right)\right)=\underline{A}. \end{array}$$

We further define the block circulant matrix bcirc(·) of any 3-way tensor $\underline{A}\in R^{n_{1}\times n_{2}\times n_{3}}$ as follows:

$$\begin{array}{c} \mathrm{bcirc}\left(\underline{A}\right):=\left[\begin{array}{cccc} A^{\left(1\right)} & \;A^{\left(n_{3}\right)} & \;\cdots & \;A^{\left(2\right)}\\ A^{\left(2\right)} & \;A^{\left(1\right)} & \;\cdots & \;A^{\left(3\right)}\\ \vdots & \;\ddots & \;\ddots & \;\vdots \\ A^{\left(n_{3}\right)} & \;A^{\left(n_{3}-1\right)} & \;\cdots & \;A^{\left(1\right)} \end{array}\right]\in C^{n_{1}n_{3}\times n_{2}n_{3}} \end{array}$$

Equipped with above defined operators, we are now in a position to define the t-product of 3-way tensors.

**Definition** **1** (t-product [33]). *Given two tensors $\underline{A}\in R^{n_{1}\times n_{2}\times n_{3}}$ and $\underline{B}\in R^{n_{2}\times n_{4}\times n_{3}}$, the t-product of $\underline{A}$ and $\underline{B}$ is a new 3-way tensor $\underline{C}$ with size $n_{1}\times n_{4}\times n_{3}$:*

(2) $$\begin{array}{c} \underline{C}=\underline{A}*\underline{B}=:\mathrm{bvfold}\left(\mathrm{bcirc}\left(\underline{A}\right)\mathrm{bvec}\left(\underline{B}\right)\right). \end{array}$$

A more intuitive interpretation of t-SVD is as follows [33]. If we treat a 3-way tensor $\underline{A}\in R^{n_{1}\times n_{2}\times n_{3}}$ as a matrix of size $n_{1}\times n_{2}$ whose entries are the tube fibers, then the tensor t-product can be analogously understood as the “matrix multiplication” where the standard scalar product is replaced with the vector circular convolution between the tubes (i.e., vectors):

(3) $$\begin{array}{c} \underline{C}=\underline{A}*\underline{B}\Leftrightarrow \underline{C}\left(i,j,:\right)=\sum_{k=1}^{n_{2}}\underline{A}\left(i,k,:\right)⋆\underline{B}\left(k,j,:\right),\;\forall i=1,2,\cdots ,n_{1},\;j=1,2,\cdots ,n_{4}, \end{array}$$

where ⋆ represent the operation of circular convolution [33] of two vectors $a,b\in R^{n_{3}}$ defined as ${\left(a⋆b\right)}_{j}=\sum_{k=1}^{n_{3}}a_{k}b_{1+\left(j-k\right)\mathrm{mod}n_{3}}$.

We also define the block diagonal matrix bdiag(·) of any 3-way tensor $\underline{A}\in R^{n_{1}\times n_{2}\times n_{3}}$ and its inverse bdfold(·) as follows:

$$\begin{array}{c} \mathrm{bdiag}\left(\underline{A}\right):=\left[\begin{array}{cc} A^{\left(1\right)} & \;\\ \ddots \\ \; & A^{\left(n_{3}\right)} \end{array}\right]\;\in R^{n_{1}n_{3}\times n_{2}n_{3}},\;\mathrm{bdfold}\left(\mathrm{bdiag}\left(\underline{A}\right)\right)=\underline{A}. \end{array}$$

We also use $\bar{A}$ (or $\bar{\underline{A}}$) to denote the block diagonal matrix of tensor $\tilde{\underline{A}}=\mathrm{dft}3\left(\underline{A}\right)$ (i.e., the Fourier version of $\underline{A}$) i.e.,

$$\begin{array}{c} \bar{A}=\mathrm{bdiag}\left(\tilde{\underline{A}}\right):=\left[\begin{array}{cc} {\tilde{A}}^{\left(1\right)} & \;\\ \ddots \\ \; & {\tilde{A}}^{\left(n_{3}\right)} \end{array}\right]\;\in C^{n_{1}n_{3}\times n_{2}n_{3}}. \end{array}$$

Then the relationship between DFT and circular convolution further indicates that the conducting t-product in the original domain is equivalent to performing standard matrix product on the Fourier block diagonal matrices [33]. Since matrix product on the Fourier block diagonal matrices can be parallel written as matrix product of all the frontal slices in the Fourier domain, we have the following relationships:

(4) $$\begin{array}{c} \underline{C}=\underline{A}*\underline{B}\Leftrightarrow \bar{C}=\bar{A}\bar{B}\Leftrightarrow {\tilde{C}}^{\left(k\right)}={\tilde{A}}^{\left(k\right)}{\tilde{B}}^{\left(k\right)},\;k=1,2,\cdots ,n_{3}. \end{array}$$

The relationship between the t-product and FFT also indicates that the inner product of two 3-way tensors $\underline{A},\underline{B}\in R^{n_{1}\times n_{2}\times n_{3}}$ and the inner product of their corresponding Fourier block diagonal matrices $\bar{\underline{A}},\bar{\underline{B}}\in C^{n_{1}n_{3}\times n_{2}n_{3}}$ satisfy the following relationship:

$$\begin{array}{c} \langle \underline{A},\underline{B}\rangle =\frac{1}{n_{3}}\langle \tilde{\underline{A}},\tilde{\underline{B}}\rangle =\frac{1}{n_{3}}\langle \bar{A},\bar{B}\rangle . \end{array}$$

When $\underline{A}=\underline{B}=\underline{X}$, one has:

$$\begin{array}{c} ∥\underline{X}{∥}_{F}=\frac{1}{\sqrt{n_{3}}}{∥\bar{\underline{X}}∥}_{F}. \end{array}$$

We further define the concepts of tensor transpose, identity tensor, f-diagonal tensor and orthogonal tensor as follows.

**Definition** **2** (tensor transpose [33]). *Given a tensor $\underline{A}\in R^{n_{1}\times n_{2}\times n_{3}}$, then define its transpose tensor ${\underline{A}}^{⊤}$ of size $n_{2}\times n_{1}\times n_{3}$ which can be formed through first transposing all the frontal slices of $\underline{A}$ and then exchanging each k-th transposed frontal slice with the $\left(n_{3}+2-k\right)$-th transposed frontal slice for all $k=2,3,\cdots ,n_{3}$.*

For example, consider 3-way tensor $\underline{A}=[A^{\left(1\right)}|A^{\left(2\right)}|A^{\left(3\right)}\left|A^{\left(4\right)}\right]\in R^{n_{1}\times n_{2}\times 4}$ with 4 frontal slices, the tensor transpose ${\underline{A}}^{⊤}$ of $\underline{A}$ is:

$$\begin{array}{c} {\underline{A}}^{⊤}=[{\left(A^{\left(1\right)}\right)}^{⊤}|{\left(A^{\left(4\right)}\right)}^{⊤}|{\left(A^{\left(3\right)}\right)}^{⊤}\left|{\left(A^{\left(2\right)}\right)}^{⊤}\right]\in R^{n_{2}\times n_{1}\times 4}. \end{array}$$

**Definition** **3** (identity tensor [33]). *The identity tensor $\underline{I}\in R^{n\times n\times n_{3}}$ is a tensor whose first frontal slice is the n-by-n identity matrix with all other frontal slices are zero matrices.*

**Definition** **4** (f-diagonal tensor [33]). *We call a 3-way tensor f-diagonal if all the frontal slices of it are diagonal matrices.*

**Definition** **5** (orthogonal tensor [33]). *We call a tensor $\underline{Q}\in R^{n\times n\times n_{3}}$ an orthogonal tensor if the following equations hold:*

$${\underline{Q}}^{⊤}*\underline{Q}=\underline{Q}*{\underline{Q}}^{⊤}=\underline{I}.$$

Then, the tensor singular value decomposition (t-SVD) can be given as follows.

**Definition** **6** (Tensor singular value decomposition, and Tensor tubal rank [38]). *Given any 3-way tensor $\underline{X}\in R^{n_{1}\times n_{2}\times n_{3}}$, then it has the following factorization called tensor singular value decomposition (t-SVD):*

(5) $$\begin{array}{c} \underline{X}=\underline{U}*\underline{\sum }*{\underline{V}}^{⊤}, \end{array}$$

*where the left and right factor tensors $\underline{U}\in R^{n_{1}\times n_{1}\times n_{3}}$ and $\underline{V}\in R^{n_{2}\times n_{2}\times n_{3}}$ are orthogonal, and the middle tensor $\underline{\sum }\in R^{n_{1}\times n_{2}\times n_{3}}$ is a rectangular f-diagonal tensor.*

**Figure 1:**
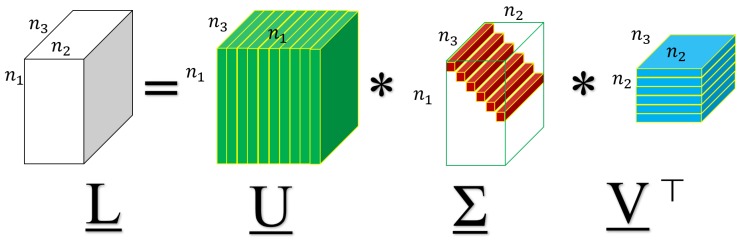
A visual illustration of t-SVD.

A visual illustration for the t-SVD is shown in Figure 1. It can be computed efficiently by FFT and IFFT in the Fourier domain according to Equation (4). For more details, see [2].

**Definition** **7** (Tensor tubal rank [38]). *The tensor tubal rank of any 3-way tensor $\underline{X}\in R^{n_{1}\times n_{2}\times n_{3}}$ is defined as the number of non-zero tubes of $\underline{\sum }$ in its t-SVD shown in Equation (5), i.e.,*

(6) $$\begin{array}{c} r_{\mathrm{tubal}}\left(\underline{A}\right):=\sum_{i}1\left(\underline{\sum }\left(i,i,:\right)\neq 0\right). \end{array}$$

**Definition** **8** (Tubal average rank [38]). *The tubal average rank $r_{a}\left(\underline{A}\right)$ of any 3-way tensor $\underline{A}\in R^{n_{1}\times n_{2}\times n_{3}}$ is defined as the averaged rank of all frontal slices of $\tilde{\underline{A}}$ as follows,*

(7) $$\begin{array}{c} r_{a}\left(\underline{A}\right):=\frac{1}{n_{3}}\sum_{k=1}^{n_{3}}\mathrm{error}\left({\tilde{A}}^{\left(k\right)}\right). \end{array}$$

**Definition** **9** (Tensor operator norm [2,38]). *The tensor operator norm $∥\underline{F}{∥}_{\mathrm{op}}$ of any 3-way tensor $\underline{F}\in R^{n_{1}\times n_{2}\times n_{3}}$ is defined as follows*:

(8) $$\begin{array}{cc} ∥\underline{F}{∥}_{\mathrm{op}}:= & \underset{∥\underline{A}{∥}_{F}\leq 1}{\sup}{∥\underline{F}*\underline{A}∥}_{F}. \end{array}$$

The relationship between t-product and FFT indicates that:

(9) $$\begin{array}{cc} ∥\underline{F}{∥}_{\mathrm{op}}:= & \underset{∥\underline{A}{∥}_{F}\leq 1}{\sup}∥\underline{F}*\underline{A}{∥}_{F}=\underset{∥\bar{A}{∥}_{F}\leq \sqrt{n_{3}}}{\sup}∥\bar{F}\cdot \bar{\underline{A}}{∥}_{F}={∥\bar{A}∥}_{\mathrm{sp}}. \end{array}$$

**Definition** **10** (Tensor spectral norm [38]). *The tensor spectral norm $∥\underline{A}{∥}_{\mathrm{sp}}$ of any 3-way tensor $\underline{F}\in R^{n_{1}\times n_{2}\times n_{3}}$ is defined as the matrix spectral norm of $\bar{A}$, i.e., *

(10) $$\begin{array}{c} ∥\underline{A}{∥}_{\mathrm{sp}}:={∥\bar{A}∥}_{\mathrm{sp}}. \end{array}$$

We further define the tubal nuclear norm.

**Definition** **11** (Tubal nuclear norm [2]). *For any tensor $\underline{A}\in R^{n_{1}\times n_{2}\times n_{3}}$ with t-SVD $\underline{A}=\underline{U}*\underline{\sum }*{\underline{V}}^{⊤}$, the tubal nuclear norm (TNN) of $\underline{A}$ is defined as*:

(11) $$\begin{array}{c} ∥\underline{A}{∥}_{\mathrm{TNN}}:=\langle \underline{\sum },\underline{I}\rangle =\sum_{i=1}^{r}\underline{\sum }\left(i,i,1\right), \end{array}$$

*where $r=r_{tubal}\left(\underline{A}\right)$.*

To understand the tubal nuclear norm, first note that:

(12) $$\begin{array}{c} r_{\mathrm{tubal}}\left(\underline{A}\right)=\sum_{i}1\left(\sum_{\_}\left(i,i,:\right)\neq 0\right)\overset{\left(i\right)}{=}\sum_{i}1\left(\tilde{\underline{\sum }}\left(i,i,:\right)\neq 0\right)\overset{\left(ii\right)}{=}\sum_{i}1(∥\tilde{\underline{\sum }}\left(i,i,:\right){∥}_{1}\neq 0)\overset{\left(iii\right)}{=}\sum_{i}1\left(\underline{\sum }\left(i,i,1\right)\neq 0\right), \end{array}$$

where (i) holds because of the definition of DFT [2], (ii) holds by the property of $l_{1}$-norm, and (iii) is a result of DFT [2]. Thus, the tubal rank of $\underline{A}$ is also the number of non-zero diagonal elements of $\underline{\sum }\left(i,i,1\right)$, i.e., the first frontal slice of tensor $\underline{\sum }$ in the t-SVD of $\underline{A}$. Similar to the matrix singular values, the values $\underline{\sum }\left(i,i,1\right),i=1,2,\cdots ,n_{3}$ are also called the singular values of tensor $\underline{A}$. As the matrix nuclear norm is the sum of matrix singular values, the tubal nuclear norm can be similarly understood as the sum of tensor singular values.

One can also verify by the property of DFT [2] that:

(13) $$\begin{array}{c} ∥\underline{A}{∥}_{\mathrm{TNN}}=\sum_{i=1}^{r}\underline{\sum }\left(i,i,1\right)=\sum_{k=1}^{n_{3}}\sum_{i=1}^{r}\tilde{\underline{\sum }}\left(i,i,k\right)=\frac{1}{n_{3}}\sum_{k=1}^{n_{3}}∥{\tilde{A}}^{\left(k\right)}{∥}_{*}=\frac{1}{n_{3}}{∥\bar{A}∥}_{*}, \end{array}$$

which indicates that the TNN of $\underline{A}\in R^{n_{1}\times n_{2}\times n_{3}}$ is also the averaged nuclear norm all frontal slices of $\tilde{\underline{A}}$. Thus, TNN indeed models the low-rankness of Fourier domain.

Now, we will show that the low-tubal-rank model is ideal to some real-world tensor data, such as color images and videos.

**Figure 2:**
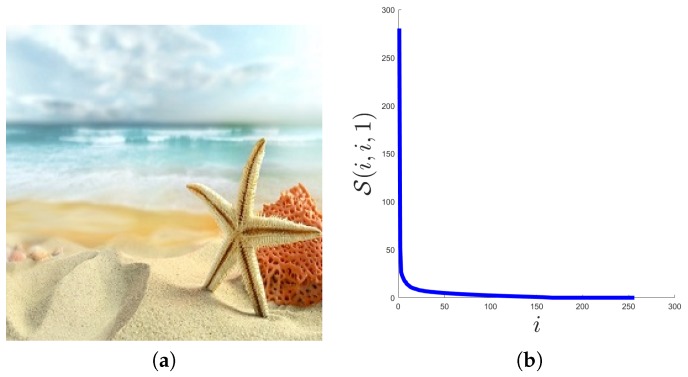
The distribution of tensor singular values $\underline{\sum }\left(i,i,1\right)$ in a natural color image. (**a**) the sample image, (**b**) the distribution of $\underline{\sum }\left(i,i,1\right)$.

First, we consider a natural image of size 256×256×3, shown in Figure 2a. In Figure 2b, we plot the distribution of its singular values, i.e., the values of $\underline{\sum }\left(i,i,1\right)$ along with the index *i*. As can be seen from Figure 2b, there are only a small number of singular values with large magnitude, and most of the singular values are close to 0. Then, we can say that some natural color images are approximately low tubal rank.

**Figure 3:**
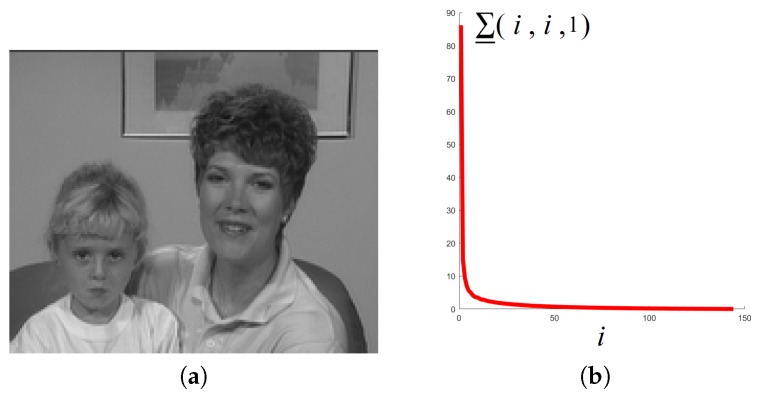
The distribution of tensor singular values $\underline{\sum }\left(i,i,1\right)$ in a video sequence. (**a**) the first frame of the video, (**b**) the distribution of $\underline{\sum }\left(i,i,1\right)$.

Then, consider a commonly used YUV sequence *Mother-daughter_qcif* (These data can be download from the following link https://sites.google.com/site/subudhibadri/fewhelpfuldownloads.) whose first frame is shown in Figure 3a. We use the Y components of the first 30 frames, and get a tensor of size 144×176×30 and show the distribution of tensor singular values in Figure 3b. We can see from Figure 3b that similar to Figure 2b, there are only a small number of singular values with large magnitude, and most of the singular values are close to 0. Then, we can say that some videos can be well approximately low tubal rank.

For TNN and tensor spectral norm, we highlight the following two lemmas.

**Lemma** **1.** *[2] TNN is the convex envelop of the tensor average rank in the unit ball of tensor spectral norm $\left\{\underline{T}\in R^{n_{1}\times n_{2}\times n_{3}}|∥\underline{T}{∥}_{\mathrm{sp}}\leq 1\right\}$.*

**Lemma** **2.** *[2] The TNN and the tensor spectral norm are dual norms to each other.*

### 2.2. Related Works

In this subsection, we briefly introduce some related works. The proposed STPCP is tightly related to the Tensor Robust Principal Component Analysis (TRPCA) which aims to recover a low-rank tensor ${\underline{L}}_{0}$ and a sparse tensor ${\underline{S}}_{0}$ from their sum $\underline{M}={\underline{L}}_{0}+{\underline{S}}_{0}$. This is a special case of our measurement Model (1) where the noise tensor ${\underline{E}}_{0}$ is a zero tensor.

In [39], the SNN-based TRPCA model is proposed by modeling the underlying tensor as a low Tucker rank one:

(14) $$\begin{array}{cc} \min_{\underline{L},\underline{S}} & \;∥\underline{L}{∥}_{\mathrm{SNN}}+{∥\underline{S}∥}_{1}\;s.t.\;\;\;\underline{L}+\underline{S}=\underline{M}, \end{array}$$

where SNN (Sum of Nuclear Norms) is defined as $∥\underline{L}{∥}_{\mathrm{SNN}}:=\sum_{i=1}^{K}\alpha_{k}{∥L_{\left(k\right)}∥}_{*},$ where $\alpha_{k}>0$ and $L_{\left(k\right)}$ is the mode-*k* matricization of $\underline{L}$ [40].

Model (14) indeed assumes the underlying tensor to be low Tucker rank, which can be too strong for some real tensor data. The TNN-based TRPCA model uses TNN to impose low-rankness in the final solution $\underline{L}$ as follows:

(15) $$\begin{array}{c} \min_{\underline{L},\underline{S}}\;∥\underline{L}{∥}_{\mathrm{TNN}}+\lambda {∥\underline{S}∥}_{1}\;\;s.t.\;\;\;\underline{L}+\underline{S}=\underline{M}. \end{array}$$

As shown in [2], when the underlying tensor ${\underline{L}}_{0}$ satisfy the tensor incoherent conditions, by solving Problem (15), one can exactly recover the underlying tensor ${\underline{L}}_{0}$ and ${\underline{S}}_{0}$ with high probability with parameter $\lambda =1/\sqrt{\max\left\{n_{1},n_{2}\right\}n_{3}}$.

When the noise tensor ${\underline{E}}_{0}$ is not zero, the robust tensor decomposition based on SNN is proposed in [36] as follows:

(16) $$\begin{array}{c} \min_{\underline{L},\underline{S}}\;\frac{1}{2}∥\underline{M}-\underline{L}-\underline{S}{∥}_{F}+\lambda_{1}∥\underline{L}{∥}_{\mathrm{SNN}}+\lambda_{2}{∥\underline{S}∥}_{1}, \end{array}$$

where $\lambda_{1}$ and $\lambda_{2}$ are positive regularization parameters. The estimation error on $\underline{L}$ and $\underline{S}$ is analyzed with an upper bound in [36].

In [37], the TNN-based RTD model is proposed as follows:

(17) $$\begin{array}{c} \min_{\underline{L},\underline{S}}\;\frac{1}{2}∥\underline{M}-\underline{L}-\underline{S}{∥}_{F}+\lambda_{1}∥\underline{L}{∥}_{\mathrm{TNN}}+\lambda_{2}∥\underline{S}{∥}_{1},\;s.t.{∥\underline{L}∥}_{\infty }\leq \alpha , \end{array}$$

where α is an upper estimate of $l_{\infty }$-norm of the underlying tensor ${\underline{L}}_{0}$. An upper bound on the estimation error is also established. However, in the analysis of Model (17), the error does not vanish as the noise tensor ${\underline{E}}_{0}$ vanishes which means the analysis cannot guarantee exact recovery in the noiseless setting (which can be provided by the analysis of TNN-based TRPCA (15) by Lu et al. [2]).

The Bayesian approach is also used for robust tensor recovery. The CP decomposition under sparse corruption and small dense noise is considered [41], and tensor rank estimation is achieved using Bayesian approach. In [42], CP decomposition under missing value and small dense noise is considered with rank estimation similar to [41]. A sparse Bayesian CP model is proposed in [43] to recover a tensor with missing value, outliers and noises. In [44], a fully Bayesian treatment is proposed to recover a low-tubal-rank tensor corrupted by both noises and outliers.

## 3. Theoretical Guarantee for Stable Tensor Principal Component Pursuit

In this section, we formulate the proposed STPCP model and give the main theoretical result which upper bounds the estimation error and guarantees exact recovery in the noiseless setting.

### 3.1. The Proposed STPCP

As for the measurement Model (1), we further assume that the noise tensor ${\underline{E}}_{0}$ has bounded energy measured in F-norm, i.e., $∥{\underline{E}}_{0}{∥}_{F}\leq \delta$. Please note that the limited energy assumption is very mild, since most signals are of limited energy.

To recover the low-rank tensor ${\underline{L}}_{0}$ and the sparse tensor ${\underline{S}}_{0}$, we first produce the following optimization problem:

(18) $$\begin{array}{cc} (\hat{\underline{L}},\hat{\underline{S}})= & \underset{\underline{L},\underline{S}}{\mathrm{argmin}}\;∥\underline{L}{∥}_{\mathrm{TNN}}+\lambda ∥\underline{S}{∥}_{1},\;s.t.\;{∥\underline{M}-\underline{L}-\underline{S}∥}_{F}\leq \delta , \end{array}$$

where λ is a positive parameter balancing the two regularizers. The motivation is to use TNN as a low-rank regularization term to exploit the low-dimensional structure in the signal tensor, whereas tensor $l_{1}$-norm is used to impose sparsity in the corruption tensor (since we assumes it to be sparse).

The relationship between Model (18) and existing models are discussed in Remark 1 and Remark 2.

**Remark** **1.** *The following models can be seen as special cases as the proposed STPCP Model (18);*

(*I*). *When δ=0, i.e., in the noiseless case, the proposed model degenerates to the TRPCA Model (15) [2].*
(*II*). *When $n_{3}=1$, then the stable tensor PCP Model (18) degenerates to the Stable Principal Component Pursuit (SPCP) [45] which aims to pursuit the principal components modeled by low-rank matrix ${\underline{L}}_{0}$ from it observation M corrupted by both noises $E_{0}$ and sparse corruptions $S_{0}$. The SPCP is formulated as follows:*
  (19) $$\begin{array}{c} \min_{L,S}{\;∥L∥}_{*}+{\lambda ∥S∥}_{1},\;s.t.\;{∥M-L-S∥}_{F}\leq \delta . \end{array}$$
(*III*). *When $n_{3}=1$ and δ=0, the proposed STPCP further degenerates to Robust Principal Component Analysis (RPCA) [46] given as follows:*
  (20) $$\begin{array}{c} \min_{L,S}{\;∥L∥}_{*}+\lambda {∥S∥}_{1},\;s.t.\;L+S=M. \end{array}$$

**Remark** **2.** *The differences from the proposed Model (18) and TNN-based RTD Model ((17) [37]) is as follows. First, our model does not need to upper estimate the $l_{\infty }$-norm of the underlying tensor. Second, our model is a constrained optimization problem, whereas Model (17) is an unconstrained optimization problem.*

### 3.2. A Theorem for Stable Recovery

To analyze the statistical performance of Model (18), we should assume on the underlying low-rank tensor ${\underline{L}}_{0}$ that it is not sparse. Only by this assumption, ${\underline{L}}_{0}$ can be identified from its mixture with sparse ${\underline{S}}_{0}$. Such an assumption can be described by the tensor incoherence condition [2,47], which is used to provide an identifiablility for low-rank ${\underline{L}}_{0}$.

**Definition** **12** (Tensor incoherence condition [2,47]). *Given a 3-way tensor $\underline{T}\in R^{n_{1}\times n_{2}\times n_{3}}$ with tubal rank r, suppose it has the skinny t-SVD $\underline{T}=\underline{U}*\underline{\Lambda }*{\underline{V}}^{⊤}$, where $\underline{U}\in R^{n_{1}\times r\times n_{3}},\underline{V}\in R^{r\times n_{2}\times n_{3}}$ are orthogonal tensors, and $\underline{\Lambda }\in R^{r\times r\times n_{3}}$ is an f-diagonal tensor. Then, $\underline{T}$ is said to satisfy the tensor incoherent condition (TIC) with parameter $\mu (\underline{T})$ if the following inequalities hold:*

(21) $$\begin{array}{cc} \max_{i\in [n_{1}]}{∥{\underline{U}}^{⊤}*{\overset{˚}{e}}_{i}∥}_{F} & \leq \sqrt{\frac{r\mu (\underline{T})}{n_{1}n_{3}}}, \end{array}$$

(22) $$\begin{array}{cc} \max_{j\in [n_{2}]}{∥{\underline{V}}^{⊤}*{\overset{˚}{e}}_{j}∥}_{F} & \leq \sqrt{\frac{r\mu (\underline{T})}{n_{2}n_{3}}}, \end{array}$$

(23) $$\begin{array}{cc} ∥\underline{U}*{\underline{V}}^{⊤}{∥}_{\infty } & \leq \sqrt{\frac{r\mu (\underline{T})}{n_{1}n_{2}n_{3}}}. \end{array}$$

*where ${\overset{˚}{e}}_{i}\in R^{n_{1}\times 1\times n_{3}}$ is a tensor column basis with only the (i,1,1)-th element being 1 and all the others being 0, and ${\overset{˚}{e}}_{j}\in R^{n_{2}\times 1\times n_{3}}$ is also a tensor column basis with only the (j,1,1)-th element being 1 and all the others being 0.*

**Assumption** **1.** *Suppose the true tensor ${\underline{L}}_{0}$ in the measurement model (1) satisfies tensor incoherence condition with parameter μ.*

Assumption 1 intrinsically ensures that the row bases and column bases of ${\underline{L}}_{0}$ do not align well with the canonical row and column bases. Thus, the low-rank ${\underline{L}}_{0}$ is not sparse, which avoids the ambiguity that low-rank component can also be sparse in the measurement Model (1).

We should also force the sparse component in Model (1) is not low rank.

**Assumption** **2.** *Assume the support *Ω* of ${\underline{S}}_{0}$ is drawn uniformly at random.*

Now we can establish an upper bound on the estimation error of $\hat{\underline{L}}$ and $\hat{\underline{S}}$ in Problem (18).

**Theorem** **1** (An Upper Bound on the Estimation Error). *Suppose ${\underline{L}}_{0}$ and ${\underline{S}}_{0}$ satisfy Assumption 1 and Assumption 2, respectively. If the tubal rank r of ${\underline{L}}_{0}$ and the sparsity (i.e., the $l_{0}$-norm) s of ${\underline{S}}_{0}$ are respectively upper bounded as follows:*

(24) $$\begin{array}{c} r\leq \frac{c_{r}\min\left\{n_{1},n_{2}\right\}}{\mu \log^{2}\left(n_{3}\max\left\{n_{1},n_{2}\right\}\right)},\;\mathrm{and}\;s\leq c_{s}n_{1}n_{2}n_{3} \end{array}$$

*where $c_{l}$ and $c_{s}$ are two sufficiently small numerical constants independent on the dimensions $n_{1}$, $n_{2}$ and $n_{3}$. Then the estimator defined in Model (18) satisfy the following inequalities:*

(25) $$\begin{array}{cc} ∥\hat{\underline{L}}-{\underline{L}}_{0}{∥}_{F} & \leq \left(\sqrt{1+\frac{1}{\max\{n_{1},n_{2}\}}}+8\left(1+2\sqrt{2}\right)\sqrt{\min\left\{n_{1},n_{2}\right\}n_{3}}\right)\delta \\ ∥\hat{\underline{S}}-{\underline{S}}_{0}{∥}_{F} & \leq \left(\sqrt{1+\max\{n_{1},n_{2}\}}+8\left(1+2\sqrt{2}\right)\sqrt{n_{1}n_{2}n_{3}}\right)\delta , \end{array}$$

*with probability at least $1-c_{1}{\left(n_{3}\max\left\{n_{1},n_{2}\right\}\right)}^{-c_{2}}$ (over the choice of support of ${\underline{S}}_{0}$), where $c_{1}$ and $c_{2}$ are positive constants independent on the dimensions $n_{1}$, $n_{2}$ and $n_{3}$.*

The proof of Theorem 1 are given in the appendix. In Theorem 1, estimation errors on ${\underline{L}}_{0}$ and ${\underline{S}}_{0}$ are separately established. It indicates that the estimation error scales linearly with the noise level δ, which is in consistence with the result in [37].

**Remark** **3.** *A significant progress over [37] is that in the noiseless setting where ${\underline{E}}_{0}$ vanishes, our analysis can provide exact recovery guarantee of ${\underline{L}}_{0}$ and ${\underline{S}}_{0}$. This is because the tensor incoherence condition adopted in our analysis intrinsically ensures that the low-rank tensor ${\underline{L}}_{0}$ is not sparse and thus can be separated from the sparse corruption tensor, whereas the non-spiky condition adopted in [37] fails to provide identifiability in the measurement Model (1).*

For Theorem 1, we also give the following remark.

**Remark** **4.** *The error bounds established in Theorem 1 are consistent with the theoretical analysis for the special cases shown in Remark 1.*

(*I*). *When δ=0, i.e., in the noiseless case, the error bounds in Theorem 1 will vanish, which means exact recovery of ${\underline{L}}_{0}$ and ${\underline{S}}_{0}$ can be guaranteed. This result is consistent with the analysis in [2] for TNN-based TRPCA Model (15).*
(*II*). *When $n_{3}=1$, the error bound on the sparse component in Theorem 1 is consistent with the error bound shown in Equation (8) of [45]. The upper bound on error of the low-rank component in Theorem 1 is sharper than that given in Equation (8) of [45].*
(*III*). *When $n_{3}=1$ and δ=0, the proposed STPCP has consistent theoretical guarantee with the analysis of RPCA [46].*

## 4. Algorithms

In this section, we design two algorithms. The first algorithm is based on the framework of ADMM [48] which has been extensively used in convex optimization with good convergence behavior. However, ADMM requires full SVDs on large matrices in each iteration which is high computational burden in high-dimensional settings. Thus, the second algorithm is proposed to solve this issue by using a factorization trick which can instead conducting SVDs on much smaller matrices.

### 4.1. An ADMM Algorithm

The proposed estimator (18) is equivalent to the following unconstrained problem:

(26) $$\begin{array}{c} \min_{\underline{L},\underline{S}}\frac{1}{2}∥\underline{L}+\underline{S}-\underline{M}{∥}_{F}^{2}+\gamma (∥\underline{L}{∥}_{\mathrm{TNN}}+\lambda ∥\underline{S}{∥}_{1}), \end{array}$$

where γ is a positive parameter balancing the data fidelity term and the regularization term.

Besides being corrupted by noises and outliers, the observed tensor $\underline{M}$ may also suffer from missing entries which can be taken as outliers with known positions in many applications. Thus, it is more practical to consider the recovery of ${\underline{L}}_{0}$ against outliers ${\underline{S}}_{0}$, noises ${\underline{E}}_{0}$ and missing entries shown in the following measurement model:

(27) $$\begin{array}{c} \underline{M}=\underline{B}⊙\left({\underline{L}}_{0}+{\underline{S}}_{0}+{\underline{E}}_{0}\right), \end{array}$$

where tensor $\underline{B}\in R^{n_{1}\times n_{2}\times n_{3}}$ denote the missing mask where ${\underline{B}}_{ijk}=1$, if the (i,j,k)-th entry of $\underline{L}$ is observed and ${\underline{B}}_{ijk}=0$ otherwise, and ⊙ denotes element-wise multiplication. Taking into consideration of missing entries, Model (26) can be further modified as:

(28) $$\begin{array}{c} \min_{\underline{L},\underline{S}}\frac{1}{2}∥\underline{B}⊙\left(\underline{L}+\underline{S}-\underline{M}\right){∥}_{F}^{2}+\gamma (∥\underline{L}{∥}_{\mathrm{TNN}}+\lambda ∥\underline{S}{∥}_{1}). \end{array}$$

By adding auxiliary variables to Problem (28), we obtain:

(29) $$\begin{array}{cc} \min_{\underline{K},\underline{L},\underline{R},\underline{S}} & \;\frac{1}{2}∥\underline{B}⊙\left(\underline{L}+\underline{S}-\underline{M}\right){∥}_{F}^{2}+\gamma ∥\underline{K}{∥}_{\mathrm{TNN}}+\gamma \lambda {∥\underline{R}∥}_{1}\\ s.t.\;\; & \;\underline{K}=\underline{L},\;\underline{R}=\underline{S}. \end{array}$$

The Augmented Lagrangian (AL) of Problem (29) is given as follows:

(30) $$\begin{array}{cc} L_{\rho }\left(\underline{L},\underline{S},\underline{K},\underline{R},{\underline{Y}}_{1},{\underline{Y}}_{2}\right)=\frac{1}{2} & ∥\underline{B}⊙\left(\underline{L}+\underline{S}-\underline{M}\right){∥}_{F}^{2}+\gamma ∥\underline{K}{∥}_{\mathrm{TNN}}+\gamma \lambda {∥\underline{R}∥}_{1}\\ & \;+\langle {\underline{Y}}_{1},\underline{K}-\underline{L}\rangle +\frac{\rho }{2}∥\underline{K}-\underline{L}{∥}_{F}^{2}+\langle {\underline{Y}}_{2},\underline{R}-\underline{S}\rangle +\frac{\rho }{2}{∥\underline{R}-\underline{S}∥}_{F}^{2}, \end{array}$$

where ${\underline{Y}}_{1},{\underline{Y}}_{2}\in R^{n_{1}\times n_{2}\times n_{3}}$ are Lagrangian multipliers and ρ is a penalty parameter.

According the strategy of ADMM, we update prime variables $\left(\underline{L},\underline{S}\right)$ and $\left(\underline{K},\underline{R}\right)$ by alternative minimization of AL in Problem (29) as follows

- Update $\left(\underline{L},\underline{S}\right)$. We update $\left(\underline{L},\underline{S}\right)$ by minimizing $L_{\rho }$ with other variables fixed as follows:
  (31) $$\begin{array}{cc} & \left({\underline{L}}^{t+1},{\underline{S}}^{t+1}\right)\\ & =\underset{\left(\underline{L},\underline{S}\right)}{\mathrm{argmin}}L_{\rho }\left(\underline{L},\underline{S},{\underline{K}}^{t},{\underline{R}}^{t},{\underline{Y}}_{1}^{t},{\underline{Y}}_{2}^{t}\right)\\ & =\underset{\left(\underline{L},\underline{S}\right)}{\mathrm{argmin}}\frac{1}{2}∥\underline{B}⊙\left(\underline{L}+\underline{S}-\underline{M}\right){∥}_{F}^{2}+\langle {\underline{Y}}_{1}^{t},{\underline{K}}^{t}-\underline{L}\rangle +\frac{\rho }{2}∥{\underline{K}}^{t}-\underline{L}{∥}_{F}^{2}+\langle {\underline{Y}}_{2}^{t},{\underline{R}}^{t}-\underline{S}\rangle +\frac{\rho }{2}{∥{\underline{R}}^{t}-\underline{S}∥}_{F}^{2}. \end{array}$$
  Taking derivatives of the right-hand side of Equation (31) with respect to $\underline{L}$ and $\underline{S}$ respectively, and setting the results zero, we obtain:
  (32) $$\begin{array}{cc} \underline{B}⊙\left({\underline{L}}^{t+1}+{\underline{S}}^{t+1}\right)-\underline{B}⊙\underline{M}-{\underline{Y}}_{1}^{t}+\rho \left({\underline{L}}^{t+1}-{\underline{K}}^{t}\right) & =\underline{0}\\ \underline{B}⊙\left({\underline{L}}^{t+1}+{\underline{S}}^{t+1}\right)-\underline{B}⊙\underline{M}-{\underline{Y}}_{2}^{t}+\rho \left({\underline{S}}^{t+1}-{\underline{R}}^{t}\right) & =\underline{0}. \end{array}$$
  Resolving the above equation group yields:
  (33) $$\begin{array}{cc} {\underline{L}}^{t+1} & =\left(\rho \left(\underline{B}+\rho \underline{1}\right)⊙{\underline{K}}^{t}+\rho \underline{B}⊙\underline{M}+\left(\underline{B}+\rho \underline{1}\right)⊙{\underline{Y}}_{1}^{t}-\underline{B}⊙{\underline{Y}}_{2}^{t}-\rho \underline{B}⊙{\underline{R}}^{t}\right)⊘\left(\rho (2\underline{B}+\rho \underline{1})\right),\\ {\underline{S}}^{t+1} & =\left(\rho \left(\underline{B}+\rho \underline{1}\right)⊙{\underline{R}}^{t}+\rho \underline{B}⊙\underline{M}+\left(\underline{B}+\rho \underline{1}\right)⊙{\underline{Y}}_{2}^{t}-\underline{B}⊙{\underline{Y}}_{1}^{t}-\rho \underline{B}⊙{\underline{K}}^{t}\right)⊘\left(\rho (2\underline{B}+\rho \underline{1})\right), \end{array}$$
  where ⊘ denotes entry-wise division and $\underline{1}$ denotes the tensor all whose entries are 1.
- Update $\left(\underline{K},\underline{R}\right)$. We update $\left(\underline{K},\underline{R}\right)$ by minimizing $L_{\rho }$ with other variables fixed as follows
  (34) $$\begin{array}{cc} & \left({\underline{K}}^{t+1},{\underline{R}}^{t+1}\right)\\ & =\underset{\left(\underline{K},\underline{R}\right)}{\mathrm{argmin}}L_{\rho }\left({\underline{L}}^{t+1},{\underline{S}}^{t+1},\underline{K},\underline{R},{\underline{Y}}_{1}^{t},{\underline{Y}}_{2}^{t}\right)\\ & =\underset{\left(\underline{K},\underline{R}\right)}{\mathrm{argmin}}\gamma ∥\underline{K}{∥}_{\mathrm{TNN}}+\gamma \lambda ∥\underline{R}{∥}_{1}+\langle {\underline{Y}}_{1}^{t},\underline{K}-{\underline{L}}^{t+1}\rangle +\frac{\rho }{2}∥\underline{K}-{\underline{L}}^{t+1}{∥}_{F}^{2}+\langle {\underline{Y}}_{2}^{t},\underline{R}-{\underline{S}}^{t+1}\rangle +\frac{\rho }{2}{∥\underline{R}-{\underline{S}}^{t+1}∥}_{F}^{2}. \end{array}$$
  Please note that Problem (34) can further be solved separately as follows:
  (35) $$\begin{array}{cc} {\underline{K}}^{t+1} & =\underset{\underline{K}}{\mathrm{argmin}}\gamma ∥\underline{K}{∥}_{\mathrm{TNN}}+\langle {\underline{Y}}_{1}^{t},\underline{K}-{\underline{L}}^{t+1}\rangle +\frac{\rho }{2}{∥\underline{K}-{\underline{L}}^{t+1}∥}_{F}^{2}\\ & =S_{\gamma \rho^{-1}}^{{∥\cdot ∥}_{\mathrm{TNN}}}\left({\underline{L}}^{t+1}-\rho^{-1}{\underline{Y}}_{1}^{t}\right). \end{array}$$
  and
  (36) $$\begin{array}{cc} {\underline{R}}^{t+1} & =\underset{\underline{R}}{\mathrm{argmin}}\gamma \lambda ∥\underline{R}{∥}_{1}+\langle {\underline{Y}}_{1}^{t},\underline{R}-{\underline{S}}^{t+1}\rangle +\frac{\rho }{2}{∥\underline{R}-{\underline{S}}^{t+1}∥}_{F}^{2}\\ & =S_{\gamma \lambda \rho^{-1}}^{{∥\cdot ∥}_{1}}\left({\underline{S}}^{t+1}-\rho^{-1}{\underline{Y}}_{2}^{t}\right), \end{array}$$
  where $S_{\tau }^{{∥\cdot ∥}_{\mathrm{TNN}}}\left(\cdot \right)$ is the proximity operator of TNN [5]. and $S_{\tau }^{{∥\cdot ∥}_{1}}\left(\cdot \right)$ is the proximity operator of tensor $l_{1}$-norm given as follows [49]:
  $$\begin{array}{cc} S_{\tau }^{{∥\cdot ∥}_{1}}\left(\underline{A}\right):= & \underset{\underline{X}}{\mathrm{argmin}}\tau ∥\underline{X}{∥}_{1}+\frac{1}{2}∥\underline{X}-\underline{A}{∥}_{F}^{2}=\mathrm{sign}\left(\underline{A}\right)⊙\max\{(|\underline{A}\left|-\tau ,0\right\}, \end{array}$$
  In [5], a closed-form expression of $S_{\tau }\left(\cdot \right)$ is given as follows:
  **Lemma** **3.** (Proximity operator of TNN [5]) *For any 3D tensor $\underline{A}\in R^{n_{1}\times n_{2}\times n_{3}}$ with reduced t-SVD $\underline{A}=\underline{U}*\underline{\Lambda }*{\underline{V}}^{⊤}$, where $\underline{U}\in R^{n_{1}\times r\times n_{3}}$ and $\underline{V}\in R^{n_{2}\times r\times n_{3}}$ are orthogonal tensors and $\underline{\Lambda }\in R^{r\times r\times n_{3}}$ is the f-diagonal tensor of singular tubes, the proximity operator $S_{\tau }^{{∥\cdot ∥}_{\mathrm{TNN}}}\left(\underline{A}\right)$ at $\underline{A}$ can be computed by:*
  $$\begin{array}{cc} S_{\tau }^{{∥\cdot ∥}_{\mathrm{TNN}}}\left(\underline{A}\right):= & \underset{\underline{X}}{\mathrm{argmin}}\tau ∥\underline{X}{∥}_{\mathrm{TNN}}+\frac{1}{2}{∥\underline{X}-\underline{A}∥}_{F}^{2}=\underline{U}*ifft3\left(\max(fft3\left(\underline{\Lambda }\right)-\tau ,0)\right)*{\underline{V}}^{⊤}, \end{array}$$
- Update $\left({\underline{Y}}_{1},{\underline{Y}}_{2}\right)$. The Lagrangian multipliers are updated by gradient ascent as follows:
  (37) $$\begin{array}{cc} {\underline{Y}}_{1}^{t+1} & ={\underline{Y}}_{1}^{t}+\rho \left({\underline{K}}^{t+1}-{\underline{L}}^{t+1}\right),\\ {\underline{Y}}_{2}^{t+1} & ={\underline{Y}}_{2}^{t}+\rho \left({\underline{R}}^{t+1}-{\underline{S}}^{t+1}\right). \end{array}$$

The algorithm is summarized in Algorithm 1. The convergence analysis of Algorithm 1 is established in Theorem 2.

**Algorithm 1** Solving Problem (29) using ADMM.
**Input:** The observed tensor $\underline{M}$, the parameters γ,λ,ρ,δ.1:Initialize t=0, ${\underline{L}}^{0}={\underline{S}}^{0}={\underline{K}}^{0}={\underline{R}}^{0}={\underline{Y}}_{1}^{0}={\underline{Y}}_{2}^{0}=\underline{0}\in R^{n_{1}\times n_{2}\times n_{3}}$2:
**for** $t=0,\cdots ,T_{\max}$ **do**
3:Update $\left({\underline{L}}^{t+1},{\underline{S}}^{t+1}\right)$ by Equation (33);4:Update $\left({\underline{K}}^{t+1},{\underline{R}}^{t+1}\right)$ by Equations (35)–(36);5:Update $\left({\underline{Y}}_{1}^{t+1},{\underline{Y}}_{2}^{t+1}\right)$ by Equation (37);6:Check the convergence criteria:
(i)convergence of variables: $∥{\underline{A}}^{t+1}-{\underline{A}}^{t}{∥}_{\infty }\leq \delta ,\;\;\forall \underline{A}\in \left\{\underline{L},\underline{S},\underline{K},\underline{R}\right\}$,(ii)convergence of constraints: $\max\{∥{\underline{K}}^{t+1}-{\underline{L}}^{t}{∥}_{\infty },∥{\underline{R}}^{t+1}-{\underline{S}}^{t+1}{∥}_{\infty }\}\leq \delta$.7:
**end for**
**Output:** $\left(\hat{\underline{L}},\hat{\underline{S}}\right)=\left({\underline{L}}^{t+1},{\underline{S}}^{t+1}\right)$.

**Theorem** **2** (Convergence of Algorithm 1). *For any ρ>0, if the unaugmented Lagrangian $L(\underline{L},\underline{S},\underline{K},\underline{R},{\underline{Y}}_{1},{\underline{Y}}_{2})$ has a saddle point, then the iterations $L({\underline{L}}^{t},{\underline{S}}^{t},{\underline{K}}^{t},{\underline{R}}^{t},{\underline{Y}}_{1}^{t},{\underline{Y}}_{2}^{t})$ in Algorithm 1 satisfy the residual convergence, objective convergence and dual variable convergence of Problem (29) as t→∞.*

The proof of Theorem 2 is given in the Appendix A.

In a single iteration of Algorithm 1, the main cost comes from updating ${\underline{L}}^{t}$ which involves computing FFT, IFFT and $n_{3}$ SVDs of $n_{1}\times n_{2}$ matrices [47]. Hence Algorithm 1 has per-iteration complexity of order $O\left(n_{1}n_{2}n_{3}\left(n_{1}\wedge n_{2}+\log n_{3}\right)\right)$. Thus, if the total iteration number is *T*, then the total computational complexity is:

(38) $$\begin{array}{c} O\left(Tn_{1}n_{2}n_{3}\left(\min\left\{n_{1},n_{2}\right\}+\log n_{3}\right)\right). \end{array}$$

### 4.2. A Faster Algorithm

To reduce the cost of computing TNN which is a main cost of Algorithm 1, we propose the following lemma which indicates that TNN is orthogonal invariant.

**Lemma** **4.** *Given a tensor $\underline{X}\in R^{r\times r\times n_{3}}$, let $\underline{Q}\in R^{n_{1}\times r\times n_{3}}$ a two semi-orthogonal tensors, i.e., ${\underline{Q}}^{⊤}*\underline{Q}=\underline{I}\in R^{r\times r\times n_{3}}$ and $r\leq \min\{n_{1},n_{2}\}$. Then, we have the following relationship:*

$$\begin{array}{c} ∥\underline{Q}*\underline{X}{∥}_{\mathrm{TNN}}={∥\underline{X}∥}_{\mathrm{TNN}}. \end{array}$$

The proof of Lemma 4 can be found in the appendix. Equipped with Lemma 4, we decompose the low-rank component in Problem (28) as follows:

$$\begin{array}{c} \underline{L}=\underline{Q}*\underline{X},\;s.t.\;\;{\underline{Q}}^{⊤}*\underline{Q}={\underline{I}}_{r}, \end{array}$$

where ${\underline{I}}_{r}\in R^{r\times r\times n_{3}}$ is an identity tensor. The similar strategy has been used in low-rank matrix recovery from gross corruptions by [50]. Furthermore, we propose the following model for Problem (28):

(39) $$\begin{array}{cc} \min_{\underline{Q},\underline{X},\underline{S}} & \;\frac{1}{2}∥\underline{B}⊙\left(\underline{Q}*\underline{X}+\underline{S}-\underline{M}\right){∥}_{F}^{2}+\gamma (∥\underline{X}{∥}_{\mathrm{TNN}}+\lambda ∥\underline{S}{∥}_{1})\\ s.t.\;\; & \;\;{\underline{Q}}^{⊤}*\underline{Q}={\underline{I}}_{r}, \end{array}$$

where *r* is an upper estimation of tubal rank of the underlying tensor $r^{*}=r_{\mathrm{tubal}}\left({\underline{L}}_{0}\right)$.

In contrast to Model (28), the proposed Model (39) is a non-convex optimization problem. That means Model (39) may have many local minima. We establish a connection between the proposed Model (39) with Model (28) in the following theorem.

**Theorem** **3** (Connection between Model (39) and Model (28)). *Let $\left({\underline{Q}}_{*},{\underline{X}}_{*},{\underline{S}}_{*}\right)$ be a global optimal solution to Problem (39). Furthermore, let $\left({\underline{L}}^{⋆},{\underline{S}}^{⋆}\right)$ be the solution to Problem (28), and $r_{\mathrm{tubal}}\left({\underline{L}}^{⋆}\right)\leq r$, where r is the initialized tubal rank. Then $\left({\underline{Q}}_{*}*{\underline{X}}_{*},{\underline{S}}_{*}\right)$ is also the optimal solution to Problem (28).*

The proof of Theorem 3 can be found in the appendix. Theorem 3 states that the global optimal point of the (non-convex) Model (39) coincides with solution of the (convex) Model (28). It further indicates that the accuracy of Model (39) cannot exceed Model (28), which can be validated numerically in the experiment section.

To solve Model (39), we also use the ADMM framework.

First, by adding auxiliary variables, we have the following problem:

(40) $$\begin{array}{cc} \min_{\underline{L},\underline{S},\underline{R},\underline{Q},\underline{X}} & \;\frac{1}{2}∥\underline{B}⊙\left(\underline{L}+\underline{S}-\underline{M}\right){∥}_{F}^{2}+\gamma (∥\underline{X}{∥}_{\mathrm{TNN}}+\lambda ∥\underline{R}{∥}_{1})\\ s.t.\;\; & \;\underline{Q}*\underline{X}=\underline{L};\;\underline{R}=\underline{S};\;{\underline{Q}}^{⊤}*\underline{Q}={\underline{I}}_{r}. \end{array}$$

The augmented Lagrangian of Problem (40) is:

(41) $$\begin{array}{cc} L_{2}^{\prime }\left(\underline{L},\underline{S},\underline{R},\underline{Q},\underline{X}\right) & =\frac{1}{2}∥\underline{B}⊙\left(\underline{L}+\underline{S}-\underline{M}\right){∥}_{F}^{2}+\gamma (∥\underline{X}{∥}_{\mathrm{TNN}}+\lambda ∥\underline{R}{∥}_{1})\\ & \;\;+\langle {\underline{Y}}_{1},\underline{Q}*\underline{X}-\underline{L}\rangle +\frac{\rho }{2}∥\underline{Q}*\underline{X}-\underline{L}{∥}_{F}^{2}+\langle {\underline{Y}}_{2},\underline{R}-\underline{S}\rangle +\frac{\rho }{2}{∥\underline{R}-\underline{S}∥}_{F}^{2}\\ & s.t.\;{\underline{Q}}^{⊤}*\underline{Q}={\underline{I}}_{r}. \end{array}$$

According the strategy of ADMM, we update prime variables $\left(\underline{L},\underline{S}\right)$ and $\left(\underline{Q},\underline{X},\underline{R}\right)$ by alternative minimization of AL in Problem (41) as follows

- Update $\left(\underline{L},\underline{S}\right)$: We update $\left(\underline{L},\underline{S}\right)$ by minimizing $L_{\rho }^{\prime }$ with other variables fixed as follows:
  (42) $$\begin{array}{cc} & \left({\underline{L}}^{t+1},{\underline{S}}^{t+1}\right)\\ & =\underset{\left(\underline{L},\underline{S}\right)}{\mathrm{argmin}}L_{\rho }^{\prime }\left(\underline{L},\underline{S},{\underline{Q}}^{t},{\underline{X}}^{t},{\underline{R}}^{t},{\underline{Y}}_{1}^{t},{\underline{Y}}_{2}^{t}\right)\\ & =\underset{\left(\underline{L},\underline{S}\right)}{\mathrm{argmin}}\frac{1}{2}∥\underline{B}⊙\left(\underline{L}+\underline{S}-\underline{M}\right){∥}_{F}^{2}+\langle {\underline{Y}}_{1}^{t},{\underline{Q}}^{t}*{\underline{X}}^{t}-\underline{L}\rangle +\frac{\rho }{2}∥{\underline{Q}}^{t}*{\underline{X}}^{t}-\underline{L}{∥}_{F}^{2}+\langle {\underline{Y}}_{2}^{t},{\underline{R}}^{t}-\underline{S}\rangle +\frac{\rho }{2}{∥{\underline{R}}^{t}-\underline{S}∥}_{F}^{2}. \end{array}$$
  Taking derivatives of the right-hand side with respect to $\underline{L}$ and $\underline{S}$ respectively, and setting the results zero, we obtain:
  (43) $$\begin{array}{cc} \underline{B}⊙\left({\underline{L}}^{t+1}+{\underline{S}}^{t+1}\right)-\underline{B}⊙\underline{M}-{\underline{Y}}_{1}^{t}+\rho \left({\underline{L}}^{t+1}-{\underline{Q}}^{t}*{\underline{X}}^{t}\right) & =\underline{0}\\ \underline{B}⊙\left({\underline{L}}^{t+1}+{\underline{S}}^{t+1}\right)-\underline{B}⊙\underline{M}-{\underline{Y}}_{2}^{t}+\rho \left({\underline{S}}^{t+1}-{\underline{R}}^{t}\right) & =\underline{0}, \end{array}$$
  Resolving the above equation group yields:
  (44) $$\begin{array}{cc} {\underline{L}}^{t+1} & =\left(\left(1+\rho \right){\underline{Q}}^{t}*{\underline{X}}^{t}+\underline{B}⊙\underline{M}+{\underline{Y}}_{1}^{t}-{\underline{R}}^{t}\right)⊘\left(2\underline{B}+\rho \underline{1}\right),\\ \;\;{\underline{S}}^{t+1} & =\left(\left(1+\rho \right){\underline{R}}^{t}+\underline{B}⊙\underline{M}+{\underline{Y}}_{2}^{t}-{\underline{Q}}^{t}*{\underline{X}}^{t}\right)⊘\left(2\underline{B}+\rho \underline{1}\right). \end{array}$$
- Update $\underline{Q}$. We update $\underline{Q}$ by minimizing $L_{\rho }^{\prime }$ with other variables fixed as follows
  (45) $$\begin{array}{c} \min_{{\underline{Q}}^{⊤}*\underline{Q}={\underline{I}}_{r}}L_{\rho }\left({\underline{L}}^{t+1},{\underline{S}}^{t+1},\underline{Q},{\underline{X}}^{t},{\underline{R}}^{t},{\underline{Y}}_{1}^{t},{\underline{Y}}_{2}^{t}\right)\\ =\min_{{\underline{Q}}^{⊤}*\underline{Q}={\underline{I}}_{r}}\langle {\underline{Y}}_{1}^{t},\underline{Q}*{\underline{X}}^{t}-{\underline{L}}^{t+1}\rangle +\frac{\rho }{2}{∥\underline{Q}*{\underline{X}}^{t}-{\underline{L}}^{t+1}∥}_{F}^{2}.\\ =\min_{{\underline{Q}}^{⊤}*\underline{Q}={\underline{I}}_{r}}\frac{\rho }{2}{∥\underline{Q}*{\underline{X}}^{t}-\left({\underline{L}}^{t+1}-\rho^{-1}{\underline{Y}}_{1}^{t}\right)∥}_{F}^{2}\\ =P\left(\left({\underline{L}}^{t+1}-\rho^{-1}{\underline{Y}}_{1}^{t}\right)*{\left({\underline{X}}^{t}\right)}^{⊤}\right), \end{array}$$
  where operator P(·) is defined in Lemma 5 as follows.
  **Lemma** **5.** ([51]) *Given any tensors $\underline{A}\in R^{r\times n_{2}\times n_{3}},\underline{B}\in R^{n_{1}\times n_{2}\times n_{3}}$, suppose tensor $\underline{B}*{\underline{A}}^{⊤}$ has t-SVD $\underline{B}*{\underline{A}}^{⊤}=\underline{U}*\underline{\Lambda }*{\underline{V}}^{⊤}$, where $\underline{U}\in R^{n_{1}\times r\times n_{3}}$ and $\underline{V}\in R^{r\times r\times n_{3}}$. Then, the problem:*
  (46) $$\begin{array}{c} \min_{{\underline{Q}}^{⊤}*\underline{Q}={\underline{I}}_{r}}{∥\underline{P}*\underline{A}-\underline{B}∥}_{F}^{2} \end{array}$$
  *has a closed-form solution as:*
  (47) $$\begin{array}{c} \underline{Q}=P\left(\underline{B}*{\underline{A}}^{⊤}\right):=\underline{U}*{\underline{V}}^{⊤}. \end{array}$$
- Update $\left(\underline{X},\underline{R}\right)$:We update $\left(\underline{X},\underline{S}\right)$ by minimizing $L_{\rho }^{\prime }$ with other variables fixed as follows:
  (48) $$\begin{array}{c} \min_{\left(\underline{X},\underline{R}\right)}L_{\rho }\left({\underline{L}}^{t+1},{\underline{S}}^{t+1},{\underline{Q}}^{t+1},\underline{X},\underline{R},{\underline{Y}}_{1}^{t},{\underline{Y}}_{2}^{t}\right)\\ =\min_{\left(\underline{X},\underline{R}\right)}\gamma ∥\underline{X}{∥}_{\mathrm{TNN}}+\gamma \lambda ∥\underline{R}{∥}_{1}+\langle {\underline{Y}}_{1}^{t},{\underline{Q}}^{t+1}*\underline{X}-{\underline{L}}^{t+1}\rangle +\frac{\rho }{2}{∥{\underline{Q}}^{t+1}*\underline{X}-{\underline{L}}^{t+1}∥}_{F}^{2}\\ \;\;\;\;+\langle {\underline{Y}}_{2}^{t},\underline{R}-{\underline{S}}^{t+1}\rangle +\frac{\rho }{2}{∥\underline{R}-{\underline{S}}^{t+1}∥}_{F}^{2}. \end{array}$$
  Please note that Problem (48) can further be solved separately as follows:
  (49) $$\begin{array}{cc} {\underline{K}}^{t+1} & =\underset{\underline{X}}{\mathrm{argmin}}\gamma ∥\underline{X}{∥}_{\mathrm{TNN}}+\langle {\underline{Y}}_{1}^{t},{\underline{Q}}^{t+1}*\underline{X}-{\underline{L}}^{t+1}\rangle +\frac{\rho }{2}{∥{\underline{Q}}^{⊤}*\underline{X}-{\underline{L}}^{t+1}∥}_{F}^{2}\\ & =\underset{\underline{X}}{\mathrm{argmin}}\gamma ∥\underline{X}{∥}_{\mathrm{TNN}}+\frac{\rho }{2}{∥{\underline{Q}}^{t+1}*\underline{X}-\left({\underline{L}}^{t+1}-\rho^{-1}{\underline{Y}}_{1}^{t}\right)∥}_{F}^{2}\\ & \overset{\left(i\right)}{=}\underset{\underline{X}}{\mathrm{argmin}}\gamma ∥\underline{X}{∥}_{\mathrm{TNN}}+\frac{\rho }{2}{∥\underline{X}-{\left({\underline{Q}}^{t+1}\right)}^{⊤}*\left({\underline{L}}^{t+1}-\rho^{-1}{\underline{Y}}_{1}^{t}\right)∥}_{F}^{2}\\ & =S_{\gamma \rho^{-1}}^{{∥\cdot ∥}_{\mathrm{TNN}}}\left({\left({\underline{Q}}^{t+1}\right)}^{⊤}*\left({\underline{L}}^{t+1}-\rho^{-1}{\underline{Y}}_{1}^{t}\right).\right) \end{array}$$
  and
  (50) $$\begin{array}{cc} {\underline{R}}^{t+1} & =\underset{\underline{R}}{\mathrm{argmin}}\gamma \lambda ∥\underline{R}{∥}_{1}+\langle {\underline{Y}}_{1}^{t},\underline{R}-{\underline{S}}^{t+1}\rangle +\frac{\rho }{2}{∥\underline{R}-{\underline{S}}^{t+1}∥}_{F}^{2}\\ & =S_{\gamma \lambda \rho^{-1}}^{{∥\cdot ∥}_{1}}\left({\underline{K}}^{t+1}-\rho^{-1}{\underline{Y}}_{2}^{t}\right). \end{array}$$
  The equality (i) in Equation (49) holds because according to ${\underline{Q}}^{⊤}*\underline{Q}=\underline{I}$, we have:
  (51) $$\begin{array}{cc} \min_{\underline{X}}{∥\underline{Q}*\underline{X}-\underline{Y}∥}_{F}^{2} & =\min_{\bar{X}}\frac{1}{n_{3}}{∥\bar{Q}\cdot \bar{X}-\bar{Y}∥}_{F}^{2}\\ & =\min_{\bar{X}}\frac{1}{n_{3}}∥\bar{Y}{∥}_{F}^{2}-\frac{2}{n_{3}}\langle \bar{Q}\cdot \bar{X},\bar{Y}\rangle +\frac{1}{n_{3}}{∥\bar{Q}\cdot \bar{X}∥}_{F}^{2}\\ & =\min_{\bar{X}}\frac{1}{n_{3}}∥\bar{Y}{∥}_{F}^{2}-\frac{2}{n_{3}}\langle \bar{X},{\bar{Q}}^{H}\bar{Y}\rangle +\frac{1}{n_{3}}{∥\bar{X}∥}_{F}^{2}\\ & =\min_{\bar{X}}\frac{1}{n_{3}}{∥\bar{X}-{\bar{Q}}^{H}\bar{Y}∥}_{F}^{2}\\ & =\min_{\underline{X}}\frac{\rho }{2}{∥\underline{X}-{\underline{Q}}^{⊤}*\underline{Y}∥}_{F}^{2}. \end{array}$$
- Update $\left({\underline{Y}}_{1},{\underline{Y}}_{2}\right)$. The Lagrangian multipliers are updated by gradient ascent as follows:
  (52) $$\begin{array}{cc} {\underline{Y}}_{1}^{t+1} & ={\underline{Y}}_{1}^{t}+\rho \left({\underline{Q}}^{t+1}*{\underline{X}}^{t+1}-{\underline{L}}^{t+1}\right),\\ {\underline{Y}}_{2}^{t+1} & ={\underline{Y}}_{2}^{t}+\rho \left({\underline{R}}^{t+1}-{\underline{S}}^{t+1}\right). \end{array}$$

The algorithmic steps are summarized in Algorithm 2. The complexity analysis is given as follows.

In each iteration of Algorithm 2, the update of $\underline{L}$ requires FFT/IFFT, and $n_{3}$ multiplications of $n_{1}$-by-*r* and *r*-by-$n_{2}$ matrices, which costs $O\left(\left(n_{1}n_{2}+rn_{1}+r_{n}2\right)n_{3}\log n_{3}+rn_{1}n_{2}n_{3}\right)$; updating $\underline{S}$ costs $O\left(n_{1}n_{2}n_{3}\right)$; updating of $\underline{Q}$ involves FFT/IFFT and $n_{3}$ SVDs of $n_{1}$-by-*r* matrices, which costs $O\left(rn_{1}n_{3}\log n_{3}+r^{2}n_{1}n_{3}\right)$; updating $\underline{X}$ involves FFT/IFFT and $n_{3}$ SVDs of *r*-by-$n_{2}$, which costs $O\left(rn_{2}n_{3}\log n_{3}+r^{2}n_{2}n_{3})\right)$. Then, the per-iteration computational complexity of Algorithm 2 is dominated by:

$$\begin{array}{c} O\left(\max\left\{n_{1}n_{2}n_{3}\log n_{3},r^{2}\left(n_{1}+n_{2}\right)n_{3}\right\}\right). \end{array}$$

Since the low-tubal-rank assumption $r\ll \min\{n_{1},n_{2}\}$ is adopted in this paper, the per-iteration of Algorithm 2 is much lower than Algorithm 1.

**Algorithm 2** Solving Problem (40) using ADMM.
**Input:** The observed tensor $\underline{M}$, an upper estimation *r* of $r_{\mathrm{tubal}}\left({\underline{L}}_{0}\right)$, the parameters γ,λ,ρ,δ.1:Initialize t=0, ${\underline{L}}^{0}={\underline{S}}^{0}={\underline{R}}^{0}={\underline{Y}}_{1}^{0}={\underline{Y}}_{2}^{0}=\underline{0}\in R^{n_{1}\times n_{2}\times n_{3}}$, ${\underline{Q}}^{0}=\underline{0}\in R^{n_{1}\times r\times n_{3}}$, ${\underline{X}}^{0}=\underline{0}\in R^{r\times n_{2}\times n_{3}}$.2:
**for** $t=0,\cdots ,T_{\max}$ **do**
3:Update $\left({\underline{L}}^{t+1},{\underline{S}}^{t+1}\right)$ by Equation (42);4:Update ${\underline{Q}}^{t+1}$ by Equation (45);5:Update $\left({\underline{X}}^{t+1},{\underline{R}}^{t+1}\right)$ by Equations (49)–(50);6:Update $\left({\underline{Y}}_{1}^{t+1},{\underline{Y}}_{2}^{t+1}\right)$ by Equation (52);7:Check the convergence criteria:(i)convergence of variables: $∥{\underline{A}}^{t+1}-{\underline{A}}^{t}{∥}_{\infty }\leq \delta ,\;\;\forall \underline{A}\in \left\{\underline{L},\underline{S},\underline{R},\underline{Q},\underline{X}\right\}$(ii)convergence of constraints: $\max\{∥{\underline{Q}}^{t+1}*{\underline{X}}^{t+1}-{\underline{L}}^{t}{∥}_{\infty },∥{\underline{R}}^{t+1}-{\underline{S}}^{t+1}{∥}_{\infty }\}\leq \delta$.8:
**end for**
**Output:** $\left(\hat{\underline{L}},\hat{\underline{S}}\right)=\left({\underline{L}}^{t+1},{\underline{S}}^{t+1}\right)$.


## 5. Experiments

### 5.1. Synthetic Data

We first verify the correctness of Theorem 1. Specifically, we check whether the following two statements indicated in Theorem 1 hold in experiments on synthetic data sets:

(I). (Exact recovery in the noiseless setting.) Our analysis guarantees that the underlying low-rank tensor ${\underline{L}}_{0}$ and sparse tensor ${\underline{S}}_{0}$ can be exactly recovered in the noiseless setting. This statement will be checked in Section 5.1.1.
(II). (Linear scaling of errors with the noise level.) In Theorem 1, the estimation errors on ${\underline{L}}_{0}$ and ${\underline{S}}_{0}$ scales linearly with the noise level δ. This statement will be checked in Section 5.1.2.

**Signal Generation.** With a given tubal rank $r_{0}$, we first generate the underlying tensor ${\underline{L}}_{0}\in R^{n_{1}\times n_{2}\times n_{3}}$ by ${\underline{L}}_{0}=\underline{A}*\underline{B}/n_{3}$, where tensors $\underline{A}\in R^{n_{1}\times r_{0}\times n_{3}}$ and $\underline{B}\in R^{r_{0}\times n_{2}\times n_{3}}$ are generated with *i.i.d.* standard Gaussian elements. Then, the sparse corruption tensor ${\underline{S}}_{0}$ is generated by choosing its support uniformly at random. The non-zero elements of ${\underline{S}}_{0}$ will be *i.i.d.* sampled from a certain distribution that will be specified afterwards. Furthermore, the noise tensor ${\underline{E}}_{0}$ is generated with entries sampled *i.i.d.* from $N(0,\sigma^{2})$ with $\sigma =c∥{\underline{L}}_{0}{∥}_{F}/\sqrt{n_{1}n_{2}n_{3}}$, where we set constant *c* is to control the signal noise ratio. Finally, the observed tensor $\underline{M}$ is formed by $\underline{M}={\underline{L}}_{0}+{\underline{S}}_{0}+{\underline{E}}_{0}$.

#### 5.1.1. Exact Recovery in the Noiseless Setting

We first check *Statement (I)*, i.e., exact recovery in the noiseless setting. Specifically, we will show that Algorithm 1 and Algorithm 2 can exactly recover the underlying tensor ${\underline{L}}_{0}$ and the sparse corruption ${\underline{S}}_{0}$. We first test the recovery performance of different tensor sizes by setting $n=n_{1}=n_{2}\in \left\{100,160,200\right\}$ and $n_{3}=20$, with $(r_{\mathrm{tubal}}\left({\underline{L}}_{0}\right),∥{\underline{S}}_{0}{∥}_{0})=\left(0.05n,0.05n^{2}n_{3}\right)$. The non-zero elements of tensor ${\underline{S}}_{0}$ is sampled from *i.i.d.* symmetric Bernoulli distribution, i.e., the possibility of being 1 or −1 are 1/2. The results are shown in Table 1. It can be seen that both Algorithm 1 and Algorithm 2 can obtain relative standard error (RSE) smaller than 1e−5 by which we can say that ${\underline{L}}_{0}$ and ${\underline{S}}_{0}$ are exact recovered. We can also see that Algorithm 2 runs much faster than Algorithm 1.

We then test whether the recovery performance can be affected by the distribution of the corruptions. This is done by choosing the non-zeros elements of ${\underline{S}}_{0}$ from *i.i.d.* standard Gaussian distribution. The experimental results are reported in Table 2. We can find that both Algorithm 1 and Algorithm 2 can exactly recover the true ${\underline{L}}_{0}$ and ${\underline{S}}_{0}$ and Algorithm 2 runs much faster than Algorithm 1.

We also conduct STPCP by Algorithm 1 and Algorithm 2 with missing entries. After generating ${\underline{L}}_{0}$, ${\underline{S}}_{0}$ and ${\underline{E}}_{0}$, we get the observation by Model (27). We choose the support of $\underline{B}$ uniformly at random with possibility 0.8 and then set elements in the chosen support to be 1. Thus, %20 of the entries are missing. The corrupted observation *M* is then formed by $\underline{M}=\underline{B}⊙\left({\underline{L}}_{0}+{\underline{S}}_{0}+{\underline{E}}_{0}\right)$. We show the recover results in Table 3. We can see that the underlying low-rank tensor ${\underline{L}}_{0}$ can be exactly recovered and the observed part of the corruption tensor $\underline{B}⊙{\underline{S}}_{0}$ can also be exactly recovered (Please note that it is impossible to recover the unobserved entries of a sparse tensor ${\underline{S}}_{0}$ [52]).

#### 5.1.2. Linear Scaling of Errors with the Noise Level

**Figure 4:**
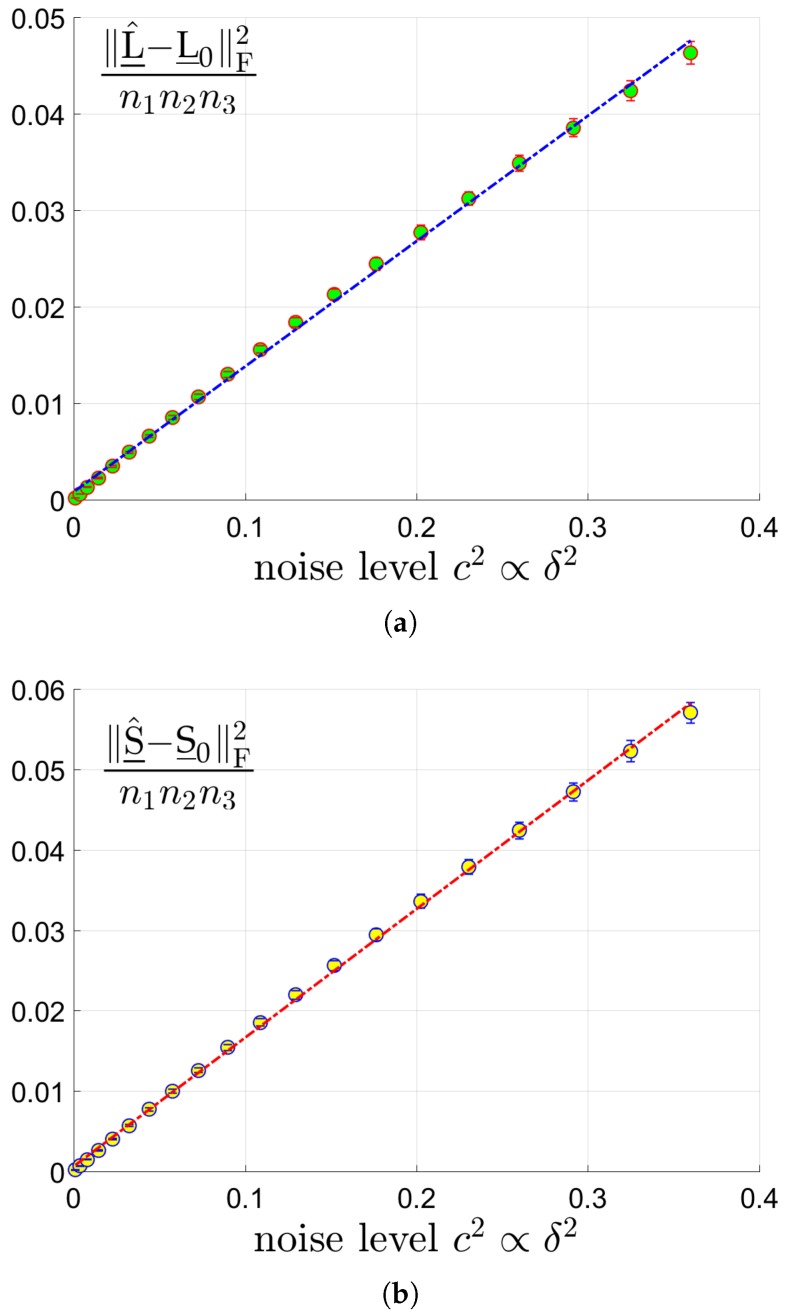
The MSEs of $\hat{\underline{L}}$ and ${\underline{S}}_{0}$ versus $c^{2}$ for tensors of size 60×60×20 where the tubal rank $r_{\mathrm{tubal}}\left({\underline{L}}_{0}\right)$ of ${\underline{L}}_{0}$ and sparsity *s* of ${\underline{S}}_{0}$ are set as $\left(r_{\mathrm{tubal}}\left({\underline{L}}_{0}\right),s\right)=\left(5,0.1n^{2}n_{3}\right)$. (**a**): MSE of $\hat{\underline{L}}$ vs $c^{2}$. (**b**): MSE of $\hat{\underline{S}}$ vs $c^{2}$.

We then verify *Statement (II)* that the estimation errors have linear scale behavior with respect to the noise level. The estimation errors are measured using the mean-squared-error (MSE):

$$\begin{array}{c} MSE\left(\hat{\underline{L}}\right)=\frac{∥\hat{\underline{L}}-{\underline{L}}_{0}{∥}_{F}^{2}}{n_{1}n_{2}n_{3}},\;MSE\left(\hat{\underline{S}}\right)=\frac{∥\hat{\underline{S}}-{\underline{S}}_{0}{∥}_{F}^{2}}{n_{1}n_{2}n_{3}}, \end{array}$$

for the low rank component ${\underline{L}}_{0}$ and the sparse component ${\underline{S}}_{0}$, respectively. We test tensors of 3 different size by choosing n∈{60,80,100} and $n_{3}=20$. The tubal rank $r_{\mathrm{tubal}}\left({\underline{L}}_{0}\right)$ of ${\underline{L}}_{0}$ and sparsity *s* of ${\underline{S}}_{0}$ are set as $\left(r_{\mathrm{tubal}}\left({\underline{L}}_{0}\right),s\right)=\left(5,0.1n^{2}n_{3}\right)$. We vary the signal noise ratio c=0.03:0.03:0.6 which is in proportional of the noise level δ. We run the proposed Algorithm 1, test 50 trials, and report the averaged MSEs. The MSEs of $\hat{\underline{L}}$ and ${\underline{S}}_{0}$ versus $c^{2}$ are shown in sub-figures (a) and (b) in Figure 4. We can see that the estimation error has linear scaling behavior along with the noise level as Theorem 1 indicates. Since the results for n=80 and n=100 are quite similar to the case of n=60, they are simply omitted.

### 5.2. Real Data Sets

In this section, experiments on real data sets (color images and videos) are carried out to evaluate the effectiveness and efficiency of the proposed Algorithms 1 and 2. Besides noises and sparse corruptions, we also consider missing values which is more challenging. The proposed algorithms are compared with the following typical models:

(I). NN-I: tensor recovery based on matrix nuclear norms of frontal slices formulated as follows:
  (53) $$\begin{array}{c} \min_{\underline{L},\underline{S}}\frac{1}{2}∥\underline{B}⊙\left(\underline{M}-\underline{L}-\underline{E}\right){∥}_{F}+\gamma \sum_{k=1}^{n_{3}}(∥L_{\left(k\right)}{∥}_{*}+\lambda ∥S^{\left(k\right)}{∥}_{1}). \end{array}$$
  This model will be used for image restoration in Section 5.2.1. Please note that Model (53) is equivalent to parallel matrix recovery on each frontal slice.
(II). NN-II: tensor recovery based on matrix nuclear norm formulated as follows:
  (54) $$\begin{array}{c} \min_{\underline{L},\underline{S}}\frac{1}{2}∥\underline{B}⊙\left(\underline{M}-\underline{L}-\underline{E}\right){∥}_{F}+{\gamma ∥L∥}_{*}+\gamma \lambda {∥\underline{S}∥}_{1}, \end{array}$$
  where $L=\left[l_{1},l_{2},\cdots ,l_{n_{3}}\right]\in R^{n_{1}n_{2}\times n_{3}}$ with $l_{k}:=\mathrm{vec}\left(L^{\left(k\right)}\right)\in R^{n_{1}n_{2}}$ defined as the vectorization [40] of frontal slices $L^{\left(k\right)}$, for all $k=1,2,\cdots ,n_{3}$. This model will be used for video restoration in Section 5.2.2.
(III). SNN: tensor recovery based on SNN formulated as follows:
  (55) $$\begin{array}{c} \min_{\underline{L},\underline{S}}\frac{1}{2}∥\underline{B}⊙\left(\underline{M}-\underline{L}-\underline{E}\right){∥}_{F}+\gamma \sum_{i=1}^{3}\alpha_{m}∥L_{\left(i\right)}{∥}_{*}+\gamma {∥\underline{S}∥}_{1}, \end{array}$$
  where $L_{\left(i\right)}\in R^{n_{i}\times \prod_{j\neq i}n_{j}}$ is the mode-*i* matriculation of tensor $\underline{L}\in R^{n_{1}\times n_{2}\times n_{3}}$, for all i=1,2,3.

We solve the above Model (53)–(55) using ADMM implemented by ourselves in Matlab. The effectiveness of the algorithms is measured by Peaks Signal Noise Ratio (PSNR):

$$\begin{array}{c} \mathrm{PSNR}:=10\log_{10}\left(\frac{n_{1}n_{2}n_{3}{∥{\underline{L}}_{0}∥}_{\infty }^{2}}{∥\hat{\underline{L}}-{\underline{L}}_{0}{∥}_{F}^{2}}\right)., \end{array}$$

Please note that a larger PSNR value indicates higher quality of $\hat{\underline{L}}$.

#### 5.2.1. Color Images

**Figure 5:**
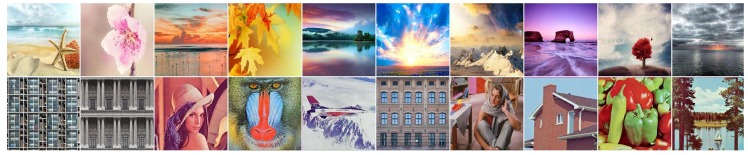
The 20 color images used.

Color images are the most commonly used 3-way tensors. We test the twenty 256-by-256-by-3 color images which have been used in [37], and carry out robust tensor recovery with missing entries (see Figure 5). Following [37], for a color image ${\underline{L}}_{0}\in R^{n\times n\times 3}$, we choose its support uniformly at random with ratio $\rho_{s}$ and fill in the values with *i.i.d.* symmetric Bernoulli variables to generate ${\underline{S}}_{0}$. The noise tensor ${\underline{E}}_{0}$ is generated with *i.i.d.* zero-mean Gaussian entries whose standard deviation is given by $\sigma =0.05∥{\underline{L}}_{0}{∥}_{F}/\sqrt{3n^{2}}$. Then, we form the binary observation mask $\underline{B}$ by choosing its support uniformly at random with ratio $\rho_{\mathrm{obs}}$. Finally, the partially observed corruption $\underline{M}=\underline{B}⊙\left({\underline{L}}_{0}+{\underline{S}}_{0}+{\underline{E}}_{0}\right)$ are formed.

**Figure 6:**
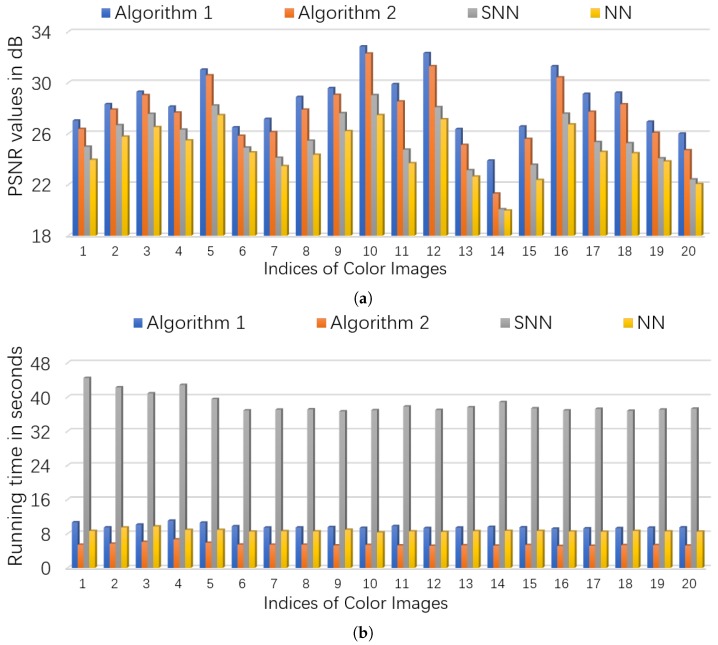
The quantitative comparison in PSNR and time on color images. First, 10% entries of each image is corrupted by *i.i.d.* symmetric Bernoulli variable, then polluted by Gaussian noise of noise level c=0.05, and finally 10% of the corrupted entries are missing uniformly at random. (**a**): the PSNR values of each algorithm; (**b**): the running time of each algorithm.

**Figure 7:**
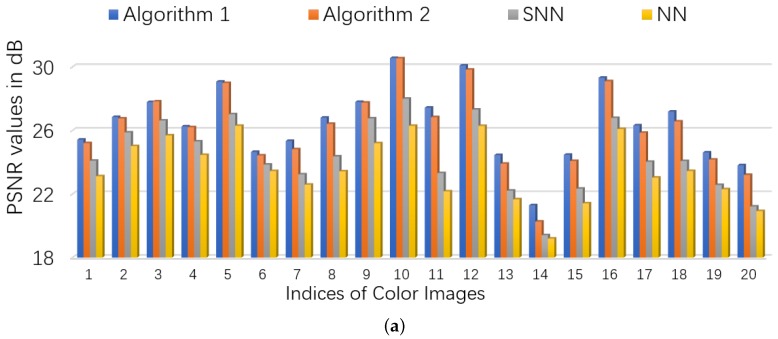

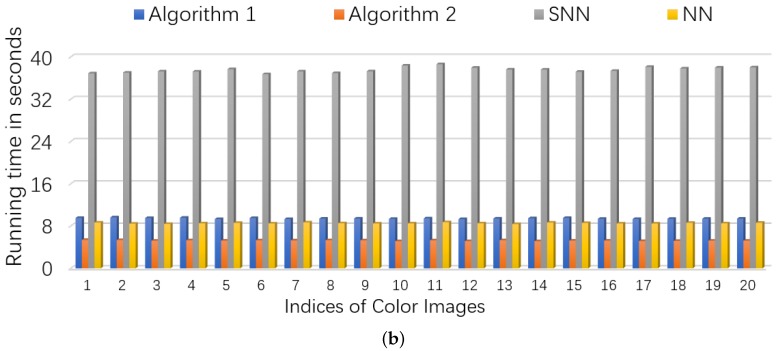
The quantitative comparison in PSNR and time on color images. First, 20% entries of each image is corrupted by *i.i.d.* symmetric Bernoulli variable, then polluted by Gaussian noise of noise level c=0.05, and finally 20% of the corrupted entries are missing uniformly at random. (**a**): the PSNR values of each algorithm; (**b**): the running time of each algorithm.

**Figure 8:**
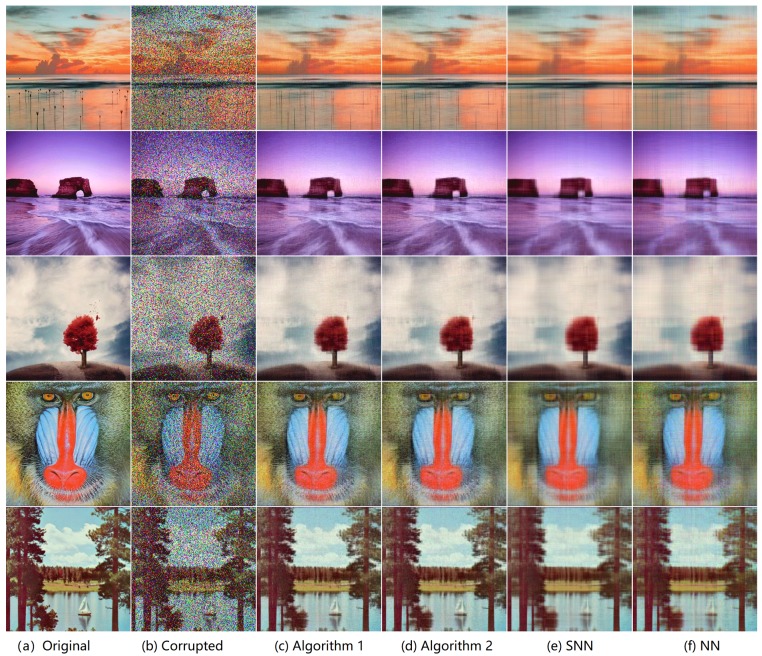
The visual results for image recovery of different algorithms. First, 20% entries of each image is corrupted by *i.i.d.* symmetric Bernoulli variable, then polluted by Gaussian noise of noise level c=0.05, and finally 20% of the corrupted entries are missing uniformly at random. (**a**): the original image; (**b**): the corrupted image; (**c**) image recovered by Algorithm 1; (**d**) image recovered by Algorithm 2; (**e**) image recovered by the matrix nuclear norm (NN)-based Model (53); (**f**) image recovered by the SNN-based Model (55).

We consider two scenarios by setting $\left(\rho_{\mathrm{obs}},\rho_{s}\right)\in \left\{\left(0.9,0.1\right),\left(0.8,0.2\right)\right\}$. For NN (Model (53)), we set the regularization parameters $\lambda =1/\sqrt{n\rho_{\mathrm{obs}}}$ (suggested by [46]), and set parameter $\gamma =∥{\underline{E}}_{0}{∥}_{\mathrm{sp}}$ where $∥{\underline{E}}_{0}{∥}_{\mathrm{sp}}$ is estimated as $6.5\sigma \sqrt{3\rho_{\mathrm{obs}}n\log\left(6n\right)}$ (suggested by [5]). For SNN, the parameters are chosen to satisfy γ=0.05, $\alpha_{1}=\alpha_{2}=\sqrt{3n\rho_{\mathrm{obs}}},\alpha_{3}=0.01\sqrt{3n\rho_{\mathrm{obs}}}$. For Algorithm 1 and Algorithm 2, we set $\gamma =0.3∥{\underline{E}}_{0}{∥}_{\mathrm{sp}}$, and $\lambda =1/\sqrt{3n\rho_{\mathrm{obs}}}$. The initialized rank *r* in Algorithm 2 is set as 60. In each setting, we test each color image for 10 times and report the averaged PSNR and time. For quantitative comparison, we show the PSNR values and running times in Figure 6 and Figure 7 for settings of $\left(\rho_{\mathrm{obs}},\rho_{s}\right)=\left(0.9,0.1\right)$ and $\left(\rho_{\mathrm{obs}},\rho_{s}\right)=\left(0.8,0.2\right)$, respectively. Several visual examples are shown in Figure 8 for qualitative comparison for the setting of $\left(\rho_{\mathrm{obs}},\rho_{s}\right)=\left(0.8,0.2\right)$. We can see from Figure 6, Figure 7 and Figure 8 that the proposed Algorithm 1 has the highest recovery quality and the proposed Algorithm 2 has the second highest quality but the fastest running time.

#### 5.2.2. Videos

In this subsection, video restoration experiments are conducted on four broadly used YUV videos (They can be downloaded from https://sites.google.com/site/subudhibadri/fewhelpfuldownloads: Akiyo_qcif, Scilent_qcif, Carphone_qcif, and Claire_qcif.) Owing to computational limitation, we simply use the first 32 frames of the Y components of all the videos which results in four 144-by-176-by-30 tensors. For a 3-way data tensor ${\underline{L}}_{0}\in R^{n_{1}\times n_{2}\times n_{3}}$, To generate corruption ${\underline{S}}_{0}$, the support is chosen uniformly at random with ratio $\rho_{s}$ and then elements in the support are filled in with *i.i.d.* symmetric Bernoulli variables. The noise tensor ${\underline{E}}_{0}$ is also generated with *i.i.d.* zero-mean Gaussian entries whose standard deviation is given by $\sigma =0.05∥{\underline{L}}_{0}{∥}_{F}/\sqrt{n_{1}n_{2}n_{3}}$. Then, the binary observation mask $\underline{B}$ is formed thorough choosing its support uniformly at random with ratio $\rho_{\mathrm{obs}}$. Finally, the partially observed corruption $\underline{M}=\underline{B}⊙\left({\underline{L}}_{0}+{\underline{S}}_{0}+{\underline{E}}_{0}\right)$ are formed.

We also consider two scenarios by setting $\left(\rho_{\mathrm{obs}},\rho_{s}\right)\in \left\{\left(0.9,0.1\right),\left(0.8,0.2\right)\right\}$. NN-II Model (54) is used in video restoration. For NN-II, we set the regularization parameters $\lambda =1/\sqrt{n_{1}n_{2}\rho_{\mathrm{obs}}}$ (suggested by [46]), and set parameter $\gamma =∥{\underline{E}}_{0}{∥}_{\mathrm{sp}}$ where $∥{\underline{E}}_{0}{∥}_{\mathrm{sp}}$ is estimated as $6.5\sigma \sqrt{\rho_{\mathrm{obs}}n_{1}n_{3}\log\left(\left(n_{1}+n_{2}\right)n_{3}\right)}$ (suggested by [5]). For SNN, the parameters are chosen to satisfy γ=0.05, $\alpha_{1}=\alpha_{2}=\sqrt{n_{1}n_{3}\rho_{\mathrm{obs}}},\alpha_{3}=5\sqrt{n_{1}n_{3}\rho_{\mathrm{obs}}}$. For Algorithm 1 and Algorithm 2, we set $\gamma =0.3∥{\underline{E}}_{0}{∥}_{\mathrm{sp}}$, and $\lambda =1/\sqrt{\max\left\{n_{1},n_{2}\right\}n_{3}\rho_{\mathrm{obs}}}$ after careful parameter tuning. The initialized rank *r* in Algorithm 2 is set as 60. In each setting, we test each video for 10 times and report the averaged PSNR and time. For quantitative comparison, we show the PSNR values and running times in Table 4. It can be seen that Algorithm 1 has the highest recovery quality and the proposed Algorithm 2 has the second highest quality but the fastest running time.

## 6. Conclusions

This paper studied the problem of stable tensor principal component pursuit which aims to recover a tensor from noises and sparse corruptions. We proposed a constrained tubal nuclear norm-based model and established upper bounds on the estimation error. In contrast to prior work [37], our theory can guarantee exact recovery in the noiseless setting. We also designed two algorithms, the first ADMM algorithm can be accelerated by the second Algorithm which adopts a factorization strategy. We validated the correctness of our theory by simulations on synthetic data, and evaluated the effectiveness and efficiency of the proposed algorithms via experiments on color images and videos.

For future directions, it is a natural and interesting extension to consider recovery of 4-way tensors [35] with arbitrary linear transformation [53,54]. It is also interesting to use tensor factorization-based methods [55,56] for STPCP. Another challenging future direction is developing tools to verify whether the unknown tensor satisfies the tensor incoherence condition from its incomplete or corrupted observations.

For extensions of the proposed approach to higher-way tensors, we produce the following two ideas:

1. By recursively applying DFT over successive modes higher than 3 and then unfolding the obtained tensor into 3-way [57], the proposed algorithms and theoretical analysis can be extended to higher-way tensors.
2. By using the overlapped orientation invariant tubal nuclear norm [58], we can extend the proposed algorithm to higher-order cases and obtain orientation invariance.

## Figures and Tables

**Table 1:** Performance of Algorithm 1 and Algorithm 2 in both accuracy and speed for different tensor sizes when the gross corruption. Outliers from symmetric Bernoulli, observation tensor $\underline{M}\in R^{n\times n\times n_{3}}$, $n_{3}=30$, $r_{\mathrm{tubal}}\left({\underline{L}}_{0}\right)=0.05n$, $∥{\underline{S}}_{0}{∥}_{1}=0.05n^{2}n_{3}$, noise level c=0, $r=\max\left\{\lfloor 2r_{\mathrm{tubal}}\left({\underline{L}}_{0}\right)\rfloor ,15\right\}$.

*n*	$r_{\mathrm{tubal}}$ ${\underline{L}}_{0}$	$∥{\underline{S}}_{0}{∥}_{0}$	Method	$r_{\mathrm{tubal}}$ $\hat{\underline{L}}$	$\frac{∥\hat{\underline{L}}-{\underline{L}}_{0}{∥}_{F}}{∥{\underline{L}}_{0}{∥}_{F}}$	$\frac{∥\hat{\underline{S}}-{\underline{S}}_{0}{∥}_{F}}{∥{\underline{S}}_{0}{∥}_{F}}$	time/s
100	5	$1\times 10^{4}$	Algorithm 1	5	$5.13\times 10^{-6}$	$5.27\times 10^{-6}$	3.63
			Algorithm 2	5	$4.92\times 10^{-6}$	$5.12\times 10^{-6}$	**1.76**
160	8	$2.56\times 10^{4}$	Algorithm 1	8	$3.86\times 10^{-6}$	$3.52\times 10^{-6}$	9.52
			Algorithm 2	8	$4.48\times 10^{-6}$	$4.08\times 10^{-6}$	**4.42**
200	10	$4\times 10^{4}$	Algorithm 1	10	$3.46\times 10^{-6}$	$3.59\times 10^{-6}$	14.16
			Algorithm 2	10	$4.12\times 10^{-6}$	$4.63\times 10^{-6}$	**7.44**

**Table 2:** Performance of Algorithm 1 and Algorithm 2 in both accuracy and speed for different tensor sizes when the gross corruption. Outliers from standard Gaussian distribution, observation tensor $\underline{M}\in R^{n\times n\times n_{3}}$, $n_{3}=30$, $r_{\mathrm{tubal}}\left({\underline{L}}_{0}\right)=0.05n$, $∥{\underline{S}}_{0}{∥}_{1}=0.05n^{2}n_{3}$, noise level c=0, $r=\max\left\{\lfloor 2r_{\mathrm{tubal}}\left({\underline{L}}_{0}\right)\rfloor ,15\right\}$.

*n*	$r_{\mathrm{tubal}}$ ${\underline{L}}_{0}$	$∥{\underline{S}}_{0}{∥}_{0}$	Method	$r_{\mathrm{tubal}}$ $\hat{\underline{L}}$	$\frac{∥\hat{\underline{L}}-{\underline{L}}_{0}{∥}_{F}}{∥{\underline{L}}_{0}{∥}_{F}}$	$\frac{∥\hat{\underline{S}}-{\underline{S}}_{0}{∥}_{F}}{∥{\underline{S}}_{0}{∥}_{F}}$	time/s
100	5	$1\times 10^{4}$	Algorithm 1	5	$2.7\times 10^{-6}$	$2.6\times 10^{-6}$	4.43
			Algorithm 2	5	$2.9\times 10^{-6}$	$3.2\times 10^{-6}$	**1.82**
160	8	$2.56\times 10^{4}$	Algorithm 1	8	$4.76\times 10^{-6}$	$4.08\times 10^{-6}$	10.45
			Algorithm 2	8	$4.24\times 10^{-6}$	$4.05\times 10^{-6}$	**5.15**
200	10	$4\times 10^{4}$	Algorithm 1	10	$3.78\times 10^{-6}$	$3.64\times 10^{-6}$	18.97
			Algorithm 2	10	$3.78\times 10^{-6}$	$3.63\times 10^{-6}$	**8.04**

**Table 3:** Performance of Algorithm 1 and Algorithm 2 in both accuracy and speed for different tensor sizes when the gross corruption. Outliers from symmetric Bernoulli, observation tensor $\underline{M}\in R^{n\times n\times n_{3}}$, $n_{3}=30$, $r_{\mathrm{tubal}}\left({\underline{L}}_{0}\right)=0.05n$, $∥{\underline{S}}_{0}{∥}_{1}=0.05n^{2}n_{3}$, noise level c=0, $r=\max\left\{\lfloor 2r_{\mathrm{tubal}}\left({\underline{L}}_{0}\right)\rfloor ,15\right\}$, with %20 random missing entries.

*n*	$r_{\mathrm{tubal}}$ ${\underline{L}}_{0}$	$∥\underline{B}⊙{\underline{S}}_{0}{∥}_{0}$	Method	$r_{\mathrm{tubal}}$ $\hat{\underline{L}}$	$\frac{∥\hat{\underline{L}}-{\underline{L}}_{0}{∥}_{F}}{∥{\underline{L}}_{0}{∥}_{F}}$	$\frac{∥\hat{\underline{S}}-\underline{B}⊙{\underline{S}}_{0}{∥}_{F}}{∥\underline{B}⊙{\underline{S}}_{0}{∥}_{F}}$	time/s
100	5	$8\times 10^{3}$	Algorithm 1	5	$7.52\times 10^{-6}$	$5.97\times 10^{-6}$	3.87
			Algorithm 2	5	$7.50\times 10^{-6}$	$5.96\times 10^{-6}$	**1.69**
160	8	$2.048\times 10^{4}$	Algorithm 1	8	$4.46\times 10^{-6}$	$5.17\times 10^{-6}$	9.64
			Algorithm 2	8	$5.60\times 10^{-6}$	$4.71\times 10^{-6}$	**4.46**
200	10	$3.2\times 10^{4}$	Algorithm 1	10	$4.78\times 10^{-6}$	$4.04\times 10^{-6}$	14.78
			Algorithm 2	10	$5.13\times 10^{-6}$	$4.20\times 10^{-6}$	**7.77**

**Table 4:** PSNR values and running time (in seconds) of different algorithms on video data. First, $\rho_{s}n_{1}n_{2}n_{3}$ entries of each image is corrupted by *i.i.d.* symmetric Bernoulli variable, then polluted by Gaussian noise of noise level c=0.05, and finally $\left(1-\rho_{\mathrm{obs}}\right)n_{1}n_{2}n_{3}$ of the corrupted entries are missing uniformly at random. The items with **highest PSNR values** are highlighted with bold face, and the items with shortest running time are highlighted with underline.

Data Set	$\left(\rho_{\mathrm{obs}},\rho_{s}\right)$	Index	NN, Model (54)	SNN, Model (55)	Algorithm 1	Algorithm 2
Akiyo	(0.9,0.1)	PSNR	31.74	32.09	**33.94**	33.36
		time/s	29.48	51.13	20.10	12.39
	(0.8,0.2)	PSNR	30.59	30.70	**32.44**	32.07
		time/s	30.65	51.17	19.53	14.92
Silent	(0.9,0.1)	PSNR	28.26	30.39	**31.74**	31.23
		time/s	28.91	49.79	21.21	14.76
	(0.8,0.2)	PSNR	26.95	27.60	**30.42**	30.07
		time/s	36.51	60.81	22.43	15.62
Carphone	(0.9,0.1)	PSNR	26.87	28.79	**29.15**	28.94
		time/s	28.55	47.17	22.12	14.41
	(0.8,0.2))	PSNR	26.12	26.43	**28.17**	27.99
		time/s	26.72	49.21	20.55	14.74
Claire	(0.9,0.1)	PSNR	30.56	32.20	**34.27**	34.02
		time/s	29.75	47.32	21.43	13.52
	(0.8,0.2)	PSNR	29.94	30.43	**32.96**	32.78
		time/s	29.43	50.46	19.47	13.04

## Appendix A. Proofs of Lemmas and Theorems

### Appendix A.1. The Proof of Theorem 1

#### Appendix A.1.1. Key Lemmas for the Proof of Theorem 1

Before Proving Theorem 1, we should define some notations and operators first.

Suppose ${\underline{L}}_{0}\in R^{n_{1}\times n_{2}\times n_{3}}$ with tubal rank *r* has the skinny t-SVD ${\underline{L}}_{0}=\underline{U}*\underline{\Lambda }*{\underline{V}}^{⊤}$, where $\underline{U}\in R^{n_{1}\times r\times n_{3}},\underline{V}\in R^{r\times n_{2}\times n_{3}}$ are orthogonal tensors, and $\underline{\Lambda }\in R^{r\times r\times n_{3}}$ is an *f*-diagonal tensor. Define the following set:

(A1) $$\begin{array}{c} T:=\left\{\underline{U}*\underline{A}+\underline{B}*{\underline{V}}^{⊤}\;|\;\underline{A}\in R^{r\times n_{2}\times n_{3}},\underline{B}\in R^{n_{1}\times r\times n_{3}}\right\}\subset R^{n_{1}\times n_{2}\times n_{3}}. \end{array}$$

Then, define the projector onto T for any tensor $\underline{T}\in R^{n_{1}\times n_{2}\times n_{3}}$ as follows:

(A2) $$\begin{array}{cc} P_{T}\left(\underline{T}\right) & :=\underline{U}*{\underline{U}}^{⊤}*\underline{T}+\underline{T}*\underline{V}*{\underline{V}}^{⊤}-\underline{U}*{\underline{U}}^{⊤}*\underline{T}*\underline{V}*{\underline{V}}^{⊤},\\ P_{T^{⊥}}\left(\underline{T}\right) & :=\left(\underline{I}-\underline{U}*{\underline{U}}^{⊤}\right)*\underline{T}*\left(\underline{I}-\underline{V}*{\underline{V}}^{⊤}\right). \end{array}$$

Let $\Omega^{⊥}$ be the complement of $\Omega \subset \left[n_{1}\right]\times \left[n_{2}\right]\times \left[n_{3}\right]$ which is the support of ${\underline{S}}_{0}$. Then, define two operators $P_{\Omega },P_{\Omega^{⊥}}$ as follows:

(A3) $$\begin{array}{c} P_{\Omega }\left(\underline{T}\right):=\sum_{(i,j,k)\in \Omega }\langle \underline{T},{\overset{˚}{e}}_{i}*{\dot{e}}_{k}*{\overset{˚}{e}}_{j}^{⊤}\rangle ,\;\;P_{\Omega^{⊥}}\left(\underline{T}\right):=\sum_{\left(i,j,k\right)\in \Omega^{⊥}}\langle \underline{T},{\overset{˚}{e}}_{i}*{\dot{e}}_{k}*{\overset{˚}{e}}_{j}^{⊤}\rangle , \end{array}$$

for any $\underline{T}\in R^{n_{1}\times n_{2}\times n_{3}}$.

Define two sets Γ and $\Gamma^{⊥}$ as follows:

(A4) $$\begin{array}{c} \Gamma =\left\{\left(\underline{A},\underline{A}\right)\;|\;\underline{A}\in R^{n_{1}\times n_{2}\times n_{3}}\right\},\;\Gamma^{⊥}=\left\{\left(\underline{A},-\underline{A}\right)\;|\;\underline{A}\in R^{n_{1}\times n_{2}\times n_{3}}\right\}. \end{array}$$

Then, for any tensors ${\underline{X}}_{\iota },{\underline{X}}_{s}\in R^{n_{1}\times n_{2}\times n_{3}}$, the projectors of the tensor $\underline{X}=\left({\underline{X}}_{\iota },{\underline{X}}_{s}\right)$ into the sets Γ and $\Gamma^{⊥}$ are given as follows, respectively:

(A5) $$\begin{array}{c} P_{\Gamma }\left(\underline{X}\right)=\left(\frac{{\underline{X}}_{\iota }+{\underline{X}}_{s}}{2},\frac{{\underline{X}}_{\iota }+{\underline{X}}_{s}}{2}\right),\;P_{\Gamma^{⊥}}\left(\underline{X}\right)=\left(\frac{{\underline{X}}_{\iota }-{\underline{X}}_{s}}{2},\frac{{\underline{X}}_{s}-{\underline{X}}_{\iota }}{2}\right). \end{array}$$

For any tensors ${\underline{X}}_{\iota },{\underline{X}}_{s}\in R^{n_{1}\times n_{2}\times n_{3}}$, define two operators on $\underline{X}=\left({\underline{X}}_{\iota },{\underline{X}}_{s}\right)$ as follows:

(A6) $$\begin{array}{c} \left(P_{T}\times P_{\Omega }\right)\left(\underline{X}\right)=\left(P_{T}\left({\underline{X}}_{\iota }\right),P_{\Omega }\left({\underline{X}}_{s}\right)\right),\;\left(P_{T^{⊥}}\times P_{\Omega^{⊥}}\right)\left(\underline{X}\right)=\left(P_{T^{⊥}}\left({\underline{X}}_{\iota }\right),P_{\Omega^{⊥}}\left({\underline{X}}_{s}\right)\right). \end{array}$$

Also define two norms as follows:

(A7) $$\begin{array}{c} ∥\underline{X}{∥}_{F}=\sqrt{∥{\underline{X}}_{\iota }{∥}_{F}^{2}+{∥{\underline{X}}_{s}∥}_{F}^{2}},\;{∥\underline{X}∥}_{F,\mu }=\sqrt{∥{\underline{X}}_{\iota }{∥}_{F}^{2}+\mu^{2}{∥{\underline{X}}_{s}∥}_{F}^{2}}. \end{array}$$

where μ is a constant that will be determined afterwards.

We first give Lemma A1 which can be seen as a modified version of Lemma C.1 in [2].

**Lemma** **A1.**
*Assume that $∥P_{\Omega }P_{T}∥\leq \frac{1}{2}$, and $\lambda \leq \frac{1}{2\sqrt{n_{3}}}$. Suppose there exists a tensor ${\underline{G}}^{*}$ satisfying the following conditions:*

(A8) $$\begin{array}{c} \left\{\begin{array}{c} P_{T}\left({\underline{G}}^{*}\right)=\underline{U}*{\underline{V}}^{⊤},\\ ∥P_{T^{⊥}}\left({\underline{G}}^{*}\right){∥}_{\mathrm{sp}}\leq \frac{1}{2},\\ ∥P_{\Omega }\left({\underline{G}}^{*}-\lambda \mathrm{sign}\left({\underline{S}}_{0}\right)\right){∥}_{F}\leq \frac{\lambda }{4},\\ ∥P_{\Omega^{⊥}}\left({\underline{G}}^{*}\right){∥}_{\infty }\leq \frac{\lambda }{2}. \end{array}\right. \end{array}$$

*Then for any perturbation $\underline{\Delta }\in R^{n_{1}\times n_{2}\times n_{3}}$, one has:*

(A9) $$\begin{array}{c} ∥{\underline{L}}_{0}+\underline{\Delta }{∥}_{\mathrm{TNN}}+\lambda {∥{\underline{S}}_{0}-\underline{\Delta }∥}_{1}\\ \geq ∥{\underline{L}}_{0}{∥}_{\mathrm{TNN}}+\lambda ∥{\underline{S}}_{0}{∥}_{1}+\left(\frac{3}{4}-{∥P_{T^{⊥}}\left(\underline{G}\right)∥}_{\mathrm{sp}}\right)∥P_{T^{⊥}}\left(\underline{\Delta }\right){∥}_{\mathrm{TNN}}+\left(\frac{3}{4}\lambda -P_{\Omega^{⊥}}(∥\underline{G}{∥}_{\infty })\right){∥P_{\Omega^{⊥}}\left(\Delta \right)∥}_{1}. \end{array}$$

**Proof.** Let ${\underline{G}}_{\iota }\in \partial {∥{\underline{L}}_{0}∥}_{\mathrm{TNN}}$, i.e., any sub-gradient of ${∥\cdot ∥}_{\mathrm{TNN}}$ at ${\underline{L}}_{0}$, then it satisfies:

(A10) $$\begin{array}{c} P_{T}\left({\underline{G}}_{\iota }\right)=\underline{U}*{\underline{V}}^{⊤},\;{∥P_{T^{⊥}}\left({\underline{G}}_{\iota }\right)∥}_{\mathrm{sp}}\leq 1. \end{array}$$

${\underline{G}}_{\iota }\in \partial {∥{\underline{L}}_{0}∥}_{\mathrm{TNN}}$ and ${\underline{G}}_{s}\in \partial (\lambda ∥{\underline{S}}_{0}{∥}_{1})$. According to the convexity of ${∥\cdot ∥}_{\mathrm{TNN}}$ and ${∥\cdot ∥}_{1}$, we have:

(A11) $$\begin{array}{c} ∥{\underline{L}}_{0}+\underline{\Delta }{∥}_{\mathrm{TNN}}\geq ∥{\underline{L}}_{0}{∥}_{\mathrm{TNN}}+\langle {\underline{G}}_{\iota },\underline{\Delta }\rangle ,\;\lambda ∥{\underline{S}}_{0}-\underline{\Delta }{∥}_{1}\geq \lambda {∥{\underline{S}}_{0}∥}_{1}-\langle {\underline{G}}_{s},\underline{\Delta }\rangle . \end{array}$$

By choosing ${\underline{G}}_{\iota }=\underline{U}*{\underline{V}}^{⊤}+\underline{P}*{\underline{Q}}^{⊤}$, where $\underline{P}$ and $\underline{Q}$ comes from the skinny t-SVD of $P_{T^{⊥}}\left(\underline{\Delta }\right)=\underline{P}*\underline{\sum }*{\underline{Q}}^{⊤}$, one has:

(A12) $$\begin{array}{cc} \langle {\underline{G}}_{\iota },\underline{\Delta }\rangle & =\langle \underline{G},\underline{\Delta }\rangle +\langle {\underline{G}}_{\iota }-\underline{G},\underline{\Delta }\rangle =\langle \underline{G},\underline{\Delta }\rangle +\langle P_{T^{⊥}}\left({\underline{G}}_{\iota }\right),P_{T^{⊥}}\left(\underline{\Delta }\right)\rangle -\langle P_{T^{⊥}}\left(\underline{G}\right),P_{T^{⊥}}\left(\underline{\Delta }\right)\rangle \\ & =\langle \underline{G},\underline{\Delta }\rangle -(1-∥P_{T^{⊥}}\left(\underline{G}\right){∥}_{\mathrm{sp}})∥P_{T^{⊥}}\left(\underline{\Delta }\right){∥}_{\mathrm{TNN}}. \end{array}$$

Also, by choosing ${\underline{G}}_{s}=\lambda sign\left({\underline{S}}_{0}\right)-sign\left(P_{\Omega^{⊥}}\left(\underline{\Delta }\right)\right)$, one has:

(A13) $$\begin{array}{cc} -\langle {\underline{G}}_{s},\underline{\Delta }\rangle & =-\langle \underline{G},\underline{\Delta }\rangle -\langle {\underline{G}}_{s}-\underline{G},\underline{\Delta }\rangle \\ & =-\langle \underline{G},\underline{\Delta }\rangle -\langle P_{\Omega }\left(\lambda sign\left({\underline{S}}_{0}\right)-\underline{G}\right),P_{\Omega }\left(\underline{\Delta }\right)\rangle -\langle P_{\Omega^{⊥}}\left({\underline{G}}_{s}\right),P_{\Omega^{⊥}}\left(\underline{\Delta }\right)\rangle +\langle P_{\Omega^{⊥}}\left(\underline{G}\right),P_{\Omega^{⊥}}\left(\underline{\Delta }\right)\rangle \\ & \geq -\langle \underline{G},\underline{\Delta }\rangle -∥P_{\Omega }\left(\lambda sign\left({\underline{S}}_{0}\right)-\underline{G}\right){∥}_{F}∥P_{\Omega }\left(\underline{\Delta }\right){∥}_{F}+∥P_{\Omega^{⊥}}\left(\underline{\Delta }\right){∥}_{1}-∥P_{\Omega^{⊥}}\left(\underline{G}\right){∥}_{\infty }{∥P_{\Omega^{⊥}}\left(\underline{\Delta }\right)∥}_{1}\\ & \geq -\langle \underline{G},\underline{\Delta }\rangle -\frac{\lambda }{4}∥P_{\Omega }\left(\underline{\Delta }\right){∥}_{F}+(1-∥P_{\Omega^{⊥}}\left(\underline{G}\right){∥}_{\infty })∥P_{\Omega^{⊥}}\left(\underline{\Delta }\right){∥}_{1} \end{array}$$

Also note that:

(A14) $$\begin{array}{cc} ∥P_{\Omega }\left(\underline{\Delta }\right){∥}_{F} & \leq ∥P_{\Omega }P_{T}\left(\underline{\Delta }\right){∥}_{F}+{∥P_{\Omega }P_{T^{⊥}}\left(\underline{\Delta }\right)∥}_{F}\\ & \leq ∥P_{\Omega }P_{T}\left(\underline{\Delta }\right){∥}_{F}+{∥P_{\Omega }P_{T^{⊥}}\left(\underline{\Delta }\right)∥}_{F}\\ & \leq \frac{1}{2}∥\underline{\Delta }{∥}_{F}+{∥P_{\Omega }P_{T^{⊥}}\left(\underline{\Delta }\right)∥}_{F}\\ & \leq \frac{1}{2}∥P_{\Omega }\left(\underline{\Delta }\right){∥}_{F}+\frac{1}{2}∥P_{\Omega^{⊥}}\left(\underline{\Delta }\right){∥}_{F}+{∥P_{\Omega }P_{T^{⊥}}\left(\underline{\Delta }\right)∥}_{F} \end{array}$$

which leads to:

(A15) $$\begin{array}{cc} ∥P_{\Omega }\left(\underline{\Delta }\right){∥}_{F} & \leq ∥P_{\Omega^{⊥}}\left(\underline{\Delta }\right){∥}_{F}+2∥P_{\Omega }P_{T^{⊥}}\left(\underline{\Delta }\right){∥}_{F}\leq ∥P_{\Omega^{⊥}}\left(\underline{\Delta }\right){∥}_{1}+2\sqrt{n_{3}}{∥P_{T^{⊥}}\left(\underline{\Delta }\right)∥}_{\mathrm{TNN}}. \end{array}$$

Putting things together, we have:

(A16) $$\begin{array}{c} ∥{\underline{L}}_{0}+\underline{\Delta }{∥}_{\mathrm{TNN}}+\lambda ∥{\underline{S}}_{0}-\underline{\Delta }{∥}_{1}-(∥{\underline{L}}_{0}{∥}_{\mathrm{TNN}}+\lambda ∥{\underline{S}}_{0}{∥}_{1})\\ \geq \left(1-\frac{\lambda \sqrt{n_{3}}}{2}-{∥P_{T^{⊥}}\left(\underline{G}\right)∥}_{\mathrm{sp}}\right)∥P_{T^{⊥}}\left(\underline{\Delta }\right){∥}_{\mathrm{TNN}}+\left(\frac{3}{4}\lambda -P_{\Omega^{⊥}}(∥\underline{G}{∥}_{\infty })\right){∥P_{\Omega^{⊥}}\left(\underline{\Delta }\right)∥}_{1}. \end{array}$$

Since $\lambda \leq \frac{1}{2\sqrt{n_{3}}}$, it holds that:

$$\begin{array}{cc} \; & ∥{\underline{L}}_{0}+\underline{\Delta }{∥}_{\mathrm{TNN}}+\lambda {∥{\underline{S}}_{0}-\underline{\Delta }∥}_{1}\\ \; & \geq ∥{\underline{L}}_{0}{∥}_{\mathrm{TNN}}+\lambda ∥{\underline{S}}_{0}{∥}_{1}+\left(\frac{3}{4}-{∥P_{T^{⊥}}\left(\underline{G}\right)∥}_{\mathrm{sp}}\right)∥P_{T^{⊥}}\left(\underline{\Delta }\right){∥}_{\mathrm{TNN}}+\left(\frac{3}{4}\lambda -P_{\Omega^{⊥}}(∥\underline{G}{∥}_{\infty })\right){∥P_{\Omega^{⊥}}\left(\Delta \right)∥}_{1}, \end{array}$$

for any perturbation $\underline{\Delta }\in R^{n_{1}\times n_{2}\times n_{3}}$. □

**Lemma** **A2.**
*Suppose that $∥P_{\Omega }P_{T}∥\leq 1/2$, then for any $\underline{X}=\left({\underline{X}}_{\iota },{\underline{X}}_{s}\right)$, we have:*

(A17) $$\begin{array}{c} ∥P_{\Gamma }\left(P_{T}\times P_{\Omega }\right)\left(\underline{X}\right){∥}_{F,\mu }^{2}\geq \frac{1+\mu^{2}}{8}{∥P_{T}\times P_{\Omega }\left(\underline{X}\right)∥}_{F}^{2}. \end{array}$$

**Proof.** According to the definitions of $P_{\Gamma }$ and $P_{T}\times P_{\Omega }$, we have:

(A18) $$\begin{array}{c} P_{\Gamma }\left(P_{T}\times P_{\Omega }\right)\left(\underline{X}\right)=\left(\frac{P_{T}\left({\underline{X}}_{\iota }\right)+P_{\Omega }\left({\underline{X}}_{s}\right)}{2},\frac{P_{T}\left({\underline{X}}_{\iota }\right)+P_{\Omega }\left({\underline{X}}_{s}\right)}{2}\right). \end{array}$$

Then, we have:

(A19) $$\begin{array}{cc} ∥P_{\Gamma }\left(P_{T}\times P_{\Omega }\right)\left(\underline{X}\right){∥}_{F,\mu }^{2} & =\left(1+\mu^{2}\right)\cdot \frac{1}{4}\cdot \left(∥P_{T}\left({\underline{X}}_{\iota }\right){∥}_{F}^{2}+{∥P_{\Omega }\left({\underline{X}}_{s}\right)∥}_{F}^{2}+2\langle P_{T}\left({\underline{X}}_{\iota }\right),P_{\Omega }\left({\underline{X}}_{s}\right)\rangle \right)\\ & \overset{}{=}\frac{\left(1+\mu^{2}\right)}{4}\left(∥P_{T}\left({\underline{X}}_{\iota }\right){∥}_{F}^{2}+{∥P_{\Omega }\left({\underline{X}}_{s}\right)∥}_{F}^{2}+2\langle P_{\Omega }P_{T}P_{T}\left({\underline{X}}_{\iota }\right),P_{\Omega }\left({\underline{X}}_{s}\right)\rangle \right)\\ & \overset{}{\geq }\frac{\left(1+\mu^{2}\right)}{4}\left(∥P_{T}\left({\underline{X}}_{\iota }\right){∥}_{F}^{2}+∥P_{\Omega }\left({\underline{X}}_{s}\right){∥}_{F}^{2}-2∥P_{\Omega }P_{T}∥∥P_{T}\left({\underline{X}}_{\iota }\right){∥}_{F}{∥P_{\Omega }\left({\underline{X}}_{s}\right)∥}_{F}\right)\\ & \overset{}{\geq }\frac{\left(1+\mu^{2}\right)}{4}\left(∥P_{T}\left({\underline{X}}_{\iota }\right){∥}_{F}^{2}+∥P_{\Omega }\left({\underline{X}}_{s}\right){∥}_{F}^{2}-2\cdot \frac{1}{2}∥P_{T}\left({\underline{X}}_{\iota }\right){∥}_{F}{∥P_{\Omega }\left({\underline{X}}_{s}\right)∥}_{F}\right)\\ & \overset{}{\geq }\frac{\left(1+\mu^{2}\right)}{4}\left(∥P_{T}\left({\underline{X}}_{\iota }\right){∥}_{F}^{2}+{∥P_{\Omega }\left({\underline{X}}_{s}\right)∥}_{F}^{2}-\frac{∥P_{T}\left({\underline{X}}_{\iota }\right){∥}_{F}^{2}+{∥P_{\Omega }\left({\underline{X}}_{s}\right)∥}_{F}^{2}}{2}\right)\\ & =\frac{\left(1+\mu^{2}\right)}{8}{∥P_{T}\times P_{\Omega }\left(\underline{X}\right)∥}_{F}^{2}. \end{array}$$

Hence completes the proof. □

#### Appendix A.1.2. Proof of Theorem 1

**Proof.** For $\underline{X}=\left(\underline{L},\underline{S}\right)$, define $∥\underline{X}{∥}_{⋄}=∥\underline{L}{∥}_{\mathrm{TNN}}+\lambda {∥\underline{S}∥}_{1}$. Let $\underline{\hat{X}}=\left(\hat{\underline{L}},\hat{\underline{S}}\right)$,${\underline{X}}^{*}=\left({\underline{L}}_{0},{\underline{S}}_{0}\right)$. According to the optimality of $\left(\hat{\underline{L}},\hat{\underline{S}}\right)$ and the feasibility of $\left({\underline{L}}_{0},{\underline{S}}_{0}\right)$, we directly have:

(A20) $$\begin{array}{cc} & ∥\underline{\hat{X}}{∥}_{⋄}\leq {∥{\underline{X}}^{*}∥}_{⋄}, \end{array}$$

(A21) $$\begin{array}{cc} & ∥\hat{\underline{L}}+\hat{\underline{S}}-\underline{M}{∥}_{F}\leq \delta , \end{array}$$

(A22) $$\begin{array}{cc} & ∥{\underline{L}}_{0}+{\underline{S}}_{0}-\underline{M}{∥}_{F}\leq \delta . \end{array}$$

Let ${\underline{\Delta }}_{\iota }=\hat{\underline{L}}-{\underline{L}}_{0}$, ${\underline{\Delta }}_{s}=\hat{\underline{S}}-{\underline{S}}_{0}$. Then, we have:

(A23) $$\begin{array}{c} ∥{\underline{\Delta }}_{\iota }+{\underline{\Delta }}_{s}{∥}_{F}=∥\hat{\underline{L}}+\hat{\underline{S}}-\underline{M}-\left({\underline{L}}_{0}+{\underline{S}}_{0}-\underline{M}\right){∥}_{F}\leq ∥\hat{\underline{L}}+\hat{\underline{S}}-\underline{M}{∥}_{F}+{∥{\underline{L}}_{0}+{\underline{S}}_{0}-\underline{M}∥}_{F}\leq 2\delta . \end{array}$$

Define the pair of error tensors $\underline{\Delta }=\underline{\hat{X}}-{\underline{X}}^{*}=\left({\underline{\Delta }}_{\iota },{\underline{\Delta }}_{s}\right)$. The goal is to bound $∥\underline{\Delta }{∥}_{F,\mu }$. First, we use the decomposition $\underline{\Delta }=P_{\Gamma }\left(\underline{\Delta }\right)+P_{\Gamma^{⊥}}\left(\underline{\Delta }\right)$, and let ${\underline{\Delta }}^{\Gamma }=P_{\Gamma }\left(\underline{\Delta }\right)=\left({\underline{\Delta }}_{\iota }^{\Gamma },{\underline{\Delta }}_{s}^{\Gamma }\right)=\left(\frac{{\underline{\Delta }}_{\iota }+{\underline{\Delta }}_{s}}{2},\frac{{\underline{\Delta }}_{\iota }+{\underline{\Delta }}_{s}}{2}\right),{\underline{\Delta }}^{\Gamma^{⊥}}=P_{\Gamma^{⊥}}\left(\underline{\Delta }\right)=\left({\underline{\Delta }}_{\iota }^{\Gamma^{⊥}},{\underline{\Delta }}_{s}^{\Gamma^{⊥}}\right)=\left(\frac{{\underline{\Delta }}_{\iota }-{\underline{\Delta }}_{s}}{2},\frac{{\underline{\Delta }}_{s}-{\underline{\Delta }}_{\iota }}{2}\right)$ for simplicity. Then, we have:

(A24) $$\begin{array}{c} ∥\underline{\Delta }{∥}_{F,\mu }=∥{\underline{\Delta }}^{\Gamma }+{\underline{\Delta }}^{\Gamma^{⊥}}{∥}_{F,\mu }\overset{}{\leq }∥{\underline{\Delta }}^{\Gamma }{∥}_{F,\mu }+{∥{\underline{\Delta }}^{\Gamma^{⊥}}∥}_{F,\mu }. \end{array}$$

Please note that ${\underline{\Delta }}_{\iota }^{\Gamma }={\underline{\Delta }}_{s}^{\Gamma }=\frac{{\underline{\Delta }}_{\iota }+{\underline{\Delta }}_{s}}{2}$, thus $∥{\underline{\Delta }}^{\Gamma }{∥}_{F,\mu }$ can be bounded easily as follows:

(A25) $$\begin{array}{c} ∥{\underline{\Delta }}^{\Gamma }{∥}_{F,\mu }=\sqrt{∥{\underline{\Delta }}_{\iota }^{\Gamma }{∥}_{F}^{2}+\mu^{2}{∥{\underline{\Delta }}_{s}^{\Gamma }∥}_{F}^{2}}=\frac{\sqrt{1+\mu^{2}}}{2}{∥{\underline{\Delta }}_{\iota }+{\underline{\Delta }}_{s}∥}_{F}\leq \delta \sqrt{1+\mu^{2}}. \end{array}$$

Then, it remains to bound $∥{\underline{\Delta }}^{\Gamma^{⊥}}{∥}_{F,\mu }$. Due to the triangular inequality:

(A26) $$\begin{array}{c} ∥{\underline{\Delta }}^{\Gamma^{⊥}}{∥}_{F,\mu }\leq ∥\left(P_{T}\times P_{\Omega }\right){\underline{\Delta }}^{\Gamma^{⊥}}{∥}_{F,\mu }+{∥\left(P_{T^{⊥}}\times P_{\Omega^{⊥}}\right){\underline{\Delta }}^{\Gamma^{⊥}}∥}_{F,\mu }, \end{array}$$

**(A) bound $∥\left(P_{T^{⊥}}\times P_{\Omega^{⊥}}\right){\underline{\Delta }}^{\Gamma^{⊥}}{∥}_{F,\mu }$**. According to the convexity of ${∥\cdot ∥}_{⋄}$ we have:

(A27) $$\begin{array}{c} ∥{\underline{X}}^{*}+\underline{\Delta }{∥}_{⋄}=∥{\underline{X}}^{*}+{\underline{\Delta }}^{\Gamma }+{\underline{\Delta }}^{\Gamma^{⊥}}{∥}_{⋄}\geq ∥{\underline{X}}^{*}+{\underline{\Delta }}^{\Gamma^{⊥}}{∥}_{⋄}-{∥{\underline{\Delta }}^{\Gamma }∥}_{⋄}. \end{array}$$

Using Lemma A1, we have:

(A28) $$\begin{array}{cc} ∥{\underline{X}}^{*}+{\underline{\Delta }}^{\Gamma^{⊥}}{∥}_{⋄} & \geq ∥{\underline{X}}^{*}{∥}_{⋄}+\left(\frac{3}{4}-{∥P_{T^{⊥}}\left(\underline{G}\right)∥}_{\mathrm{sp}}\right)∥P_{T^{⊥}}\left({\underline{\Delta }}_{\iota }^{\Gamma^{⊥}}\right){∥}_{\mathrm{TNN}}+\left(\frac{3}{4}\lambda -P_{\Omega^{⊥}}(∥\underline{G}{∥}_{\infty })\right){∥P_{\Omega^{⊥}}\left({\underline{\Delta }}_{s}^{\Gamma^{⊥}}\right)∥}_{1}\\ & \geq ∥{\underline{X}}^{*}{∥}_{⋄}+\frac{1}{4}{∥\left(P_{T^{⊥}}\times P_{\Omega^{⊥}}\right){\underline{\Delta }}^{\Gamma^{⊥}}∥}_{⋄}. \end{array}$$

Combining Equations (A20), (A27) and (A28), we have:

(A29) $$\begin{array}{c} ∥{\underline{\Delta }}^{\Gamma }{∥}_{⋄}\geq \frac{1}{4}{∥\left(P_{T^{⊥}}\times P_{\Omega^{⊥}}\right){\underline{\Delta }}^{\Gamma^{⊥}}∥}_{⋄} \end{array}$$

Then, with $\mu =\sqrt{n_{3}}\lambda$, we reach a bound on $∥\left(P_{T^{⊥}}\times P_{\Omega^{⊥}}\right){\underline{\Delta }}^{\Gamma^{⊥}}{∥}_{F,\mu }$ as follows:

(A30) $$\begin{array}{cc} ∥\left(P_{T^{⊥}}\times P_{\Omega^{⊥}}\right){\underline{\Delta }}^{\Gamma^{⊥}}{∥}_{F,\mu } & \leq ∥P_{T^{⊥}}\left({\underline{\Delta }}_{\iota }^{\Gamma^{⊥}}\right){∥}_{F}+\mu {∥P_{\Omega^{⊥}}\left({\underline{\Delta }}_{s}^{\Gamma^{⊥}}\right)∥}_{F}\\ & \leq \sqrt{n_{3}}∥P_{T^{⊥}}\left({\underline{\Delta }}_{\iota }^{\Gamma^{⊥}}\right){∥}_{\mathrm{TNN}}+\mu {∥P_{\Omega^{⊥}}\left({\underline{\Delta }}_{s}^{\Gamma^{⊥}}\right)∥}_{1}\\ & \overset{}{\leq }\sqrt{n_{3}}\left(∥P_{T^{⊥}}\left({\underline{\Delta }}_{\iota }^{\Gamma^{⊥}}\right){∥}_{\mathrm{TNN}}+\lambda {∥P_{\Omega^{⊥}}\left({\underline{\Delta }}_{s}^{\Gamma^{⊥}}\right)∥}_{1}\right)\\ & \leq \sqrt{n_{3}}{∥\left(P_{T^{⊥}}\times P_{\Omega^{⊥}}\right){\underline{\Delta }}^{\Gamma^{⊥}}∥}_{⋄}\\ & \overset{}{\leq }4\sqrt{n_{3}}{∥{\underline{\Delta }}^{\Gamma }∥}_{⋄}\\ & \leq 4\sqrt{n_{3}}\left(∥{\underline{\Delta }}_{\iota }^{\Gamma }{∥}_{\mathrm{TNN}}+\lambda {∥{\underline{\Delta }}_{s}^{\Gamma }∥}_{1}\right)\\ & \overset{}{\leq }4\sqrt{n_{3}}\left(\sqrt{\min\{n_{1},n_{2}\}}∥{\underline{\Delta }}_{\iota }^{\Gamma }{∥}_{F}+\lambda \sqrt{n_{1}n_{2}n_{3}}{∥{\underline{\Delta }}_{s}^{\Gamma }∥}_{F}\right)\\ & \overset{}{=}4\sqrt{n_{3}}\left(\sqrt{\min\{n_{1},n_{2}\}}+\lambda \sqrt{n_{1}n_{2}n_{3}}\right){∥{\underline{\Delta }}_{\iota }^{\Gamma }∥}_{F}\\ & \overset{}{\leq }4\sqrt{n_{3}}\left(\sqrt{\min\{n_{1},n_{2}\}}+\lambda \sqrt{n_{1}n_{2}n_{3}}\right)\delta . \end{array}$$

**(B) bound $∥\left(P_{T}\times P_{\Omega }\right){\underline{\Delta }}^{\Gamma^{⊥}}{∥}_{F,\mu }$**. Please note that:

(A31) $$\begin{array}{c} P_{\Gamma }\left({\underline{\Delta }}^{\Gamma^{⊥}}\right)\overset{}{=}\underline{0}=P_{\Gamma }\left(P_{T}\times P_{\Omega }\right)\left({\underline{\Delta }}^{\Gamma^{⊥}}\right)+P_{\Gamma }\left(P_{T^{⊥}}\times P_{\Omega^{⊥}}\right)\left({\underline{\Delta }}^{\Gamma^{⊥}}\right), \end{array}$$

which means:

(A32) $$\begin{array}{cc} ∥P_{\Gamma }\left(P_{T}\times P_{\Omega }\right)\left({\underline{\Delta }}^{\Gamma^{⊥}}\right){∥}_{F,\mu } & =∥P_{\Gamma }\left(P_{T^{⊥}}\times P_{\Omega^{⊥}}\left({\underline{\Delta }}^{\Gamma^{⊥}}\right)\right){∥}_{F,\mu }\overset{}{\leq }{∥P_{T^{⊥}}\times P_{\Omega^{⊥}}\left({\underline{\Delta }}^{\Gamma^{⊥}}\right)∥}_{F,\mu }. \end{array}$$

According to Lemma A2, we have:

(A33) $$\begin{array}{cc} ∥\left(P_{T}\times P_{\Omega }\right)\left({\underline{\Delta }}^{\Gamma^{⊥}}\right){∥}_{F,\mu } & \overset{}{\leq }∥P_{T}\left({\underline{\Delta }}_{\iota }^{\Gamma^{⊥}}\right){∥}_{F}+\mu {∥P_{\Omega }\left({\underline{\Delta }}_{\iota }^{\Gamma^{⊥}}\right)∥}_{F}\\ & \overset{}{\leq }\sqrt{1+\mu^{2}}\sqrt{∥P_{T}\left({\underline{\Delta }}_{\iota }^{\Gamma^{⊥}}\right){∥}_{F}^{2}+{∥P_{\Omega }\left({\underline{\Delta }}_{s}^{\Gamma^{⊥}}\right)∥}_{F}^{2}}\\ & \overset{}{=}\sqrt{1+\mu^{2}}{∥P_{T}\times P_{\Omega }\left({\underline{\Delta }}^{\Gamma^{⊥}}\right)∥}_{F}\\ & \overset{}{\leq }\sqrt{1+\mu^{2}}\cdot \sqrt{\frac{8}{\sqrt{1+\mu^{2}}}}\cdot {∥P_{\Gamma }\left(P_{T}\times P_{\Omega }\right)\left({\underline{\Delta }}^{\Gamma^{⊥}}\right)∥}_{F}. \end{array}$$

According to Equations (A32) and (A33), we obtain:

(A34) $$\begin{array}{c} ∥\left(P_{T}\times P_{\Omega }\right)\left({\underline{\Delta }}^{\Gamma^{⊥}}\right){∥}_{F,\mu }\leq 2\sqrt{2}{∥\left(P_{T^{⊥}}\times P_{\Omega^{⊥}}\right)\left({\underline{\Delta }}^{\Gamma^{⊥}}\right)∥}_{F,\mu }. \end{array}$$

Thus, combing Equations (A24), (A25), (A30) and (A34), and setting $\mu =\sqrt{n_{3}}\lambda$, we obtain:

(A35) $$\begin{array}{c} ∥\underline{\Delta }{∥}_{F,\mu }\leq \left(\sqrt{1+n_{3}\lambda^{2}}+4\left(1+2\sqrt{2}\right)\left(\sqrt{\min\left\{n_{1},n_{2}\right\}n_{3}}+n_{3}\lambda \sqrt{n_{1}n_{2}}\right)\right)\delta . \end{array}$$

Since $\lambda =\frac{1}{\sqrt{\max\left\{n_{1},n_{2}\right\}n_{3}}}$, we have:

(A36) $$\begin{array}{c} ∥\underline{\Delta }{∥}_{F,\mu }\leq \left(\sqrt{1+\frac{1}{\max\{n_{1},n_{2}\}}}+8\left(1+2\sqrt{2}\right)\sqrt{\min\left\{n_{1},n_{2}\right\}n_{3}}\right)\delta , \end{array}$$

which indicates that:

(A37) $$\begin{array}{cc} ∥\hat{\underline{L}}-{\underline{L}}_{0}{∥}_{F} & \leq \left(\sqrt{1+\frac{1}{\max\{n_{1},n_{2}\}}}+8\left(1+2\sqrt{2}\right)\sqrt{\min\left\{n_{1},n_{2}\right\}n_{3}}\right)\delta \\ ∥\hat{\underline{S}}-{\underline{S}}_{0}{∥}_{F} & \leq \left(\sqrt{1+\max\{n_{1},n_{2}\}}+8\left(1+2\sqrt{2}\right)\sqrt{n_{1}n_{2}n_{3}}\right)\delta . \end{array}$$

Moreover, according to the analysis in [2], the conditions $∥P_{\Omega }P_{T}∥\leq \frac{1}{2}$ and Equation (A8) in Lemma A1 hold with probability at least $1-c_{1}{\left(n_{3}\max\left\{n_{1},n_{2}\right\}\right)}^{-c_{2}}$, where $c_{1}$ and $c_{2}$ are positive constants. In this way, the proof of Theorem 1 is completed. □

### Appendix A.2. Proof of Theorem 2

**Proof.** The key idea is to rewrite Problem (29) into a standard two-block ADMM problem. For notational simplicity, let:

$$\begin{array}{c} f\left(x\right)=\frac{1}{2}∥\underline{L}+\underline{S}-\underline{Y}{∥}_{F}^{2},\;\;g\left(z\right)=\gamma {∥\underline{K}∥}_{\mathrm{TNN}}+\gamma \lambda R\left(\underline{S}\right), \end{array}$$

where x,y,z and A are defined as follows:

$$\begin{array}{c} x=\left[\begin{array}{c} \mathrm{vec}(\underline{L})\\ \mathrm{vec}(\underline{S}) \end{array}\right],\;\;y=\left[\begin{array}{c} \mathrm{vec}({\underline{Y}}_{1})\\ \mathrm{vec}({\underline{Y}}_{2}) \end{array}\right],\;\;z=\left[\begin{array}{c} \mathrm{vec}(\underline{K})\\ \mathrm{vec}(\underline{R}) \end{array}\right],\;\;A=\left[\begin{array}{cc} \mathrm{diag}(\mathrm{vec}\left(\underline{B}\right)) & 0\\ 0 & \mathrm{diag}(\mathrm{vec}\left(\underline{B}\right)) \end{array}\right], \end{array}$$

and vec(·) denotes an operation of tensor vectorization (see [40]). It can be verified that f(·) and g(·) are closed, proper convex functions. Then, Problem (29) can be re-written as follows:

$$\begin{array}{cc} \min_{x,z}\; & \;f(x)+g(z)\\ s.t.\;\;\; & \;Ax-z=0. \end{array}$$

According to the convergence analysis in [48], we have:

$$\begin{array}{cccc} \; & \mathrm{objective}\;\mathrm{convergence}: & \; & \lim_{t\to \infty }f\left(x^{t}\right)+g\left(z^{t}\right)=f^{⋆}+g^{⋆},\\ \; & \mathrm{dual}\;\mathrm{variable}\;\mathrm{convergence}: & \; & \lim_{t\to \infty }y^{t}=y^{⋆},\\ \; & \mathrm{constraint}\;\mathrm{convergence}: & \; & \lim_{t\to \infty }Ax^{t}-z^{t}=0, \end{array}$$

where $f^{⋆},g^{⋆}$ are the optimal values of f(x), g(z), respectively. Variable $y^{⋆}$ is a dual optimal point defined as:

$$\begin{array}{c} y^{⋆}=\left[\begin{array}{c} \mathrm{vec}({\underline{Y}}_{1}^{⋆})\\ \mathrm{vec}({\underline{Y}}_{2}^{⋆}) \end{array}\right], \end{array}$$

where $\left({\underline{Y}}_{1}^{⋆},{\underline{Y}}_{2}^{⋆}\right)$ is the dual component of a saddle point $\left({\underline{L}}^{⋆},{\underline{S}}^{⋆},{\underline{K}}^{⋆},{\underline{R}}^{⋆},{\underline{Y}}_{1}^{⋆},{\underline{Y}}_{2}^{⋆}\right)$ of the unaugmented Lagrangian $L(\underline{L},\underline{S},\underline{K},\underline{R},{\underline{Y}}_{1},{\underline{Y}}_{2})$. □

### Appendix A.3. Proof of Lemma 4

**Proof.** Let the full t-SVD of $\underline{X}$ be $\underline{X}=\underline{U}*\underline{\Lambda }*{\underline{V}}^{⊤}$, where $\underline{U},\underline{V}\in R^{r\times r\times n_{3}}$ are orthogonal tensors and $\underline{\Lambda }\in R^{r\times r\times n_{3}}$ is *f*-diagonal. Then:

(A38) $$\begin{array}{cc} ∥\underline{X}{∥}_{\mathrm{TNN}} & =∥\bar{\underline{U}*\underline{\Lambda }*{\underline{V}}^{⊤}}{∥}_{*}=∥\bar{\underline{U}}\cdot \bar{\underline{\Lambda }}\cdot \bar{{\underline{V}}^{⊤}}{∥}_{*}=∥\bar{\underline{\Lambda }}{∥}_{*}. \end{array}$$

Then $\underline{Q}*\underline{X}=\left(\underline{Q}*\underline{U}\right)*\underline{\Lambda }*{\underline{V}}^{⊤}$. Since

(A39) $$\begin{array}{cc} {\left(\underline{Q}*\underline{U}\right)}^{⊤}*\left(\underline{Q}*\underline{U}\right) & ={\underline{U}}^{⊤}*{\underline{Q}}^{⊤}*\underline{Q}*\underline{U}=\underline{I}, \end{array}$$

we obtain that:

(A40) $$\begin{array}{cc} ∥\underline{Q}*\underline{X}{∥}_{\mathrm{TNN}} & =∥\bar{\underline{Q}*\underline{X}}{∥}_{*}\\ & =∥\bar{\left(\underline{Q}*\underline{U}\right)*\underline{\Lambda }{\underline{V}}^{⊤}}{∥}_{*}\\ & =∥\bar{\left(\underline{Q}*\underline{U}\right)}\cdot \bar{\underline{\Lambda }}\cdot \bar{{\underline{V}}^{⊤}}{∥}_{*}\\ & =∥\bar{\underline{\Lambda }}{∥}_{*}. \end{array}$$

Thus, $∥\underline{Q}*\underline{X}{∥}_{\mathrm{TNN}}={∥\underline{X}∥}_{\mathrm{TNN}}$. □

### Appendix A.4. Proof of Theorem 3

**Proof.** Please note that $\left({\underline{Q}}_{*}*{\underline{X}}_{*},{\underline{S}}_{*}\right)$ is a feasible point of Problem (28), then we have:

(A41) $$\begin{array}{c} \frac{1}{2}∥\underline{B}⊙\left({\underline{L}}^{⋆}+{\underline{S}}^{⋆}-\underline{M}\right){∥}_{F}^{2}+\gamma (∥{\underline{L}}^{⋆}{∥}_{\mathrm{TNN}}+\lambda ∥{\underline{S}}^{⋆}{∥}_{1})\\ \leq \frac{1}{2}∥\underline{B}⊙\left({\underline{Q}}_{*}*{\underline{X}}_{*}+{\underline{S}}_{*}-\underline{M}\right){∥}_{F}^{2}+\gamma (∥{\underline{Q}}_{*}*{\underline{X}}_{*}{∥}_{\mathrm{TNN}}+\lambda ∥{\underline{S}}_{*}{∥}_{1})\\ =\frac{1}{2}∥\underline{B}⊙\left({\underline{Q}}_{*}*{\underline{X}}_{*}+{\underline{S}}_{*}-\underline{M}\right){∥}_{F}^{2}+\gamma (∥{\underline{X}}_{*}{∥}_{\mathrm{TNN}}+\lambda ∥{\underline{S}}_{*}{∥}_{1}) \end{array}$$

By the assumption that $r_{\mathrm{tubal}}\left({\underline{L}}^{⋆}\right)\leq r$, there exists a decomposition ${\underline{L}}^{⋆}={\underline{Q}}^{⋆}*{\underline{X}}^{⋆}$, such that $\left({\underline{Q}}^{⋆},{\underline{X}}^{⋆},{\underline{S}}^{⋆}\right)$ is also a feasible point of Problem (39). Moreover, since $\left({\underline{Q}}_{*},{\underline{X}}_{*},{\underline{S}}_{*}\right)$ is a global optimal solution to Problem (39), then we have that

$$\begin{array}{c} \frac{1}{2}∥\underline{B}⊙\left({\underline{Q}}_{*}*{\underline{X}}_{*}+{\underline{S}}_{*}-\underline{M}\right){∥}_{F}^{2}+\gamma (∥{\underline{X}}_{*}{∥}_{\mathrm{TNN}}+\lambda ∥{\underline{S}}_{*}{∥}_{1})\\ \leq \frac{1}{2}∥\underline{B}⊙\left({\underline{Q}}^{⋆}*{\underline{X}}^{⋆}+{\underline{S}}^{⋆}-\underline{M}\right){∥}_{F}^{2}+\gamma (∥{\underline{X}}^{⋆}{∥}_{\mathrm{TNN}}+\lambda ∥{\underline{S}}^{⋆}{∥}_{1}). \end{array}$$

By ${\underline{L}}^{⋆}={\underline{Q}}^{⋆}*{\underline{X}}^{⋆}$, we have:

(A42) $$\begin{array}{c} ∥{\underline{L}}^{⋆}{∥}_{\mathrm{TNN}}=∥{\underline{Q}}^{⋆}*{\underline{X}}^{⋆}{∥}_{\mathrm{TNN}}={∥{\underline{X}}^{⋆}∥}_{\mathrm{TNN}}. \end{array}$$

Thus, we deduce:

(A43) $$\begin{array}{c} \frac{1}{2}∥\underline{B}⊙\left({\underline{Q}}_{*}*{\underline{X}}_{*}+{\underline{S}}_{*}-\underline{M}\right){∥}_{F}^{2}+\gamma (∥{\underline{X}}_{*}{∥}_{\mathrm{TNN}}+\lambda ∥{\underline{S}}_{*}{∥}_{1})\\ \leq \frac{1}{2}∥\underline{B}⊙\left({\underline{L}}^{⋆}+{\underline{S}}^{⋆}-\underline{M}\right){∥}_{F}^{2}+\gamma (∥{\underline{L}}^{⋆}{∥}_{\mathrm{TNN}}+\lambda ∥{\underline{S}}^{⋆}{∥}_{1}). \end{array}$$

According to Equations (A41) and (A43), we further have:

(A44) $$\begin{array}{c} \frac{1}{2}∥\underline{B}⊙\left({\underline{Q}}_{*}*{\underline{X}}_{*}+{\underline{S}}_{*}-\underline{M}\right){∥}_{F}^{2}+\gamma (∥{\underline{X}}_{*}{∥}_{\mathrm{TNN}}+\lambda ∥{\underline{S}}_{*}{∥}_{1})\\ \leq \frac{1}{2}∥\underline{B}⊙\left({\underline{L}}^{⋆}+{\underline{S}}^{⋆}-\underline{M}\right){∥}_{F}^{2}+\gamma (∥{\underline{L}}^{⋆}{∥}_{\mathrm{TNN}}+\lambda ∥{\underline{S}}^{⋆}{∥}_{1}). \end{array}$$

In this way, $\left({\underline{Q}}_{*}*{\underline{X}}_{*},{\underline{S}}_{*}\right)$ is also the optimal solution to Problem (28). □
